# *In situ* characterization of post-synthetic metalation in porous salt thin films

**DOI:** 10.1039/d4sc08061k

**Published:** 2025-07-29

**Authors:** Joe D. Simmons, Subham Sarkar, Andrew A. Ezazi, Aishanee Sur, Ethan T. Iverson, Merissa N. Morey, Austin D. Chivington, Sarah G. Fisher, Jaime C. Grunlan, David C. Powers, Eric D. Bloch

**Affiliations:** a Department of Chemistry, Indiana University Bloomington IN 47405 USA edbloch@iu.edu; b Department of Chemistry, Texas A&M University College Station Texas 77843 USA powers@chem.tamu.edu; c Department of Mechanical Engineering, Texas A&M University College Station Texas 77843 USA; d Department of Material Science and Engineering, Texas A&M University College Station Texas 77843 USA

## Abstract

Post-synthetic metalation and metathesis chemistry are central to rational synthesis of metal–organic frameworks (MOFs) that are unavailable by direct self-assembly. The inherent microcrystallinity and heterogeneous nature of many MOFs renders characterization of the rate, extent, and distribution of post-synthetic modifications challenging. Here we describe the deposition of optically transparent, permanently porous thin films comprised of peripherally carboxylated free-base porphyrins and cationic porous molecular cages. The films are assembled *via* layer-by-layer growth controlled by coulombic charge pairing, which allows for systematic control over film thickness. The obtained thin films are optically transparent monoliths that retain the permanent porosity of the corresponding porous salts. Post-synthetic metalation of these films with Mn(HMDS)_2_ affords the corresponding Mn(ii) porphyrin-based materials (HMDS = hexamethyldisilazide). Access to thin films with systematically varied thickness (and thus optical density), combined with *in situ* spectroscopy, enables the kinetics and extent of metalation to be directly monitored. We demonstrate both structure- and thickness-dependence on metalation kinetics. These results provide a unique window into the molecular-scale mechanisms that underpin materials synthesis.

## Abstract 2

Controlled growth of porous, optically transparent thin films reveals structure- and thickness-dependent effects and enables *in situ* study of post-synthetic metalation kinetics.

## Graphical abstract

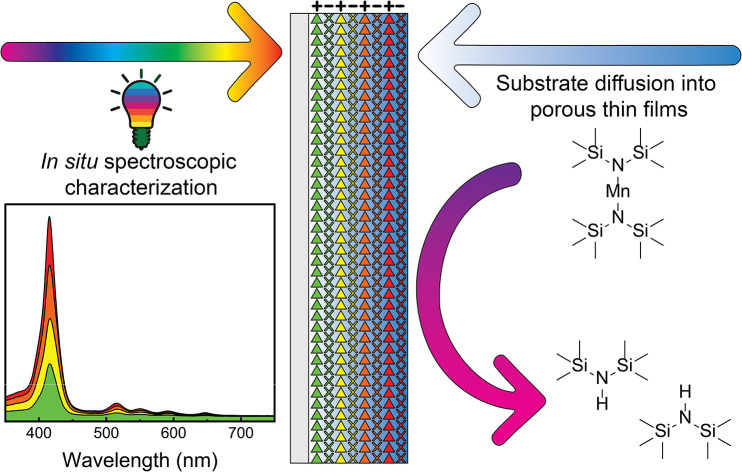

## Introduction

Metal–organic frameworks (MOFs) provide the opportunity to import concepts of molecular coordination chemistry to the solid state by virtue of (1) the predictable geometries of lattice ions and specifically designed polytopic linkers,^1^ and (2) the availability of post-synthetic methods that enable installation of geometrically defined metal ions with specific functionality not inherent to the parent framework structure.^2–8^ The synthetic modularity enables the direct incorporation of photoactive moieties such as porphyrins into the MOFs and related materials, including covalent organic frameworks (COFs) and porous organic polymers (POPs).^9–13^ This combined with material porosity, has motivated ongoing interest in utilizing these materials as platforms for catalysis and photocatalysis.^14,15^ At the same time, post-synthetic functionalization of MOFs remains a poorly understood phenomenon.^16^ For example, post-synthetic metalation of MOFs often results in core–shell materials due to localization of modifications near crystallite surfaces.^1^ Characterization of the growth and post-synthetic processes that give rise to reactive MOFs is challenging due to intrinsic material heterogeneity.^17^ Limited light penetration and scattering effects in (micro)crystalline samples limit application of *in situ* optical spectroscopy to surface phenomena when assessing the extent of metalation, the distribution of reactive metal sites, and kinetics of these processes.^18^

Optically transparent thin films provide opportunities to apply *in situ* spectroscopic tools to obtain real-time monitoring of solid-state reactions.^19,20^ While many approaches – drop casting,^21^ vacuum sublimation,^22^ and others^23–25^ have been advanced for the preparation of thin films of photoactive molecules, including conjugated macrocycles such as porphyrins, the precision of layer-by-layer growth varies and control over thickness and composition can be challenging. More specialized methods such as thermal-evaporation^26,27^ can make homogeneous, well-defined films, but the technique falls short for films comprised of multiple phases and is limited to volatile, thermally stable precursors. Electrostatic deposition methods include layer-by-layer growth of alternating charged phases. Wrighton *et al.* reported this for photoactive species with the deposition of insoluble ion-paired porphyrins.^28^ The resulting films were optically transparent and could be deposited on unmodified surfaces. These molecular deposition methods, however, produce nonporous materials.

We recently disclosed porous salts as a family of porous materials that are assembled *via* non-specific coulombic interactions instead of the covalent linkages that characterize MOFs.^29,30^ Further, we demonstrated that reactive porous salts,^31^ comprised of organometallic ions and permanently porous molecular cages of complementary charge, can be deposited as optically transparent, porous films.^32^ We reasoned that thin films based on typical MOF components or related structures would enable direct analysis of growth and post-synthetic modification processes; access to high aspect ratio architectures of porous materials that are compatible with *in situ* transmission mode spectroscopy would allow characterization of the bulk film material rather than just the surface. To realize the potential of these optically transparent architectures for both solid-state photochemistry and as platforms to study material assembly and post-synthetic modification, detailed understanding of the deposition and growth mechanism and variables are needed.

**Fig. 1:**
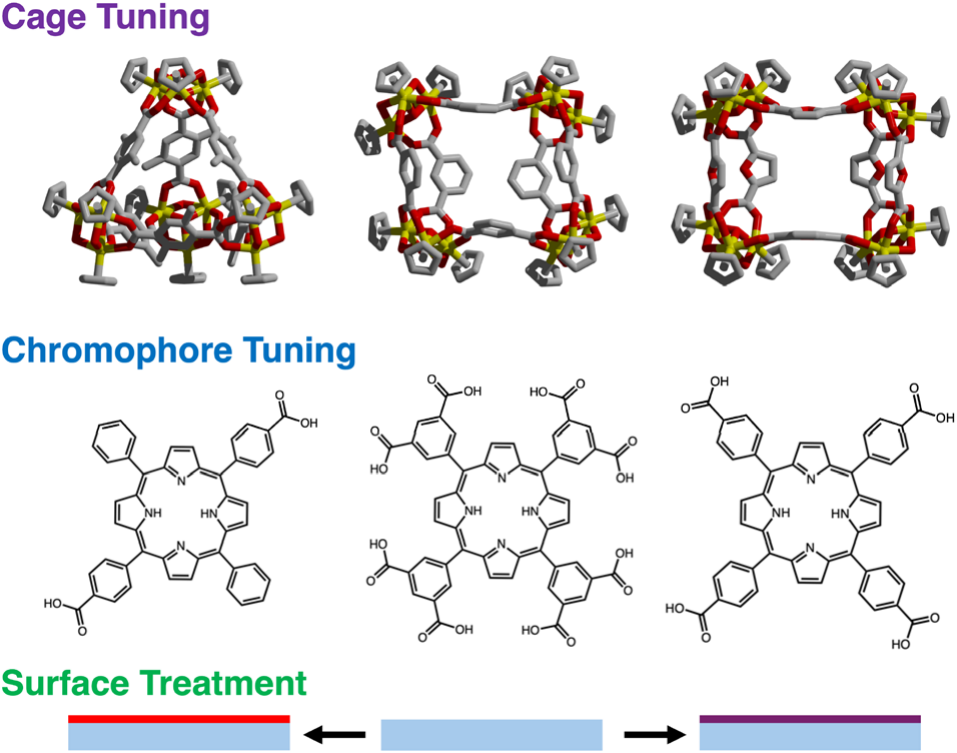
Optically transparent porous salt thin films are highly tunable as their bulk properties can be modified by judicious selection of porous cage (top), chromophore (middle), and surface treatment, including several factors such as plasma etching and silylation (bottom).

Here, we describe a detailed study of the mechanism of film deposition and post-synthetic metalation. First, we elucidate the impact of molecular charge, cage structure, and surface chemistry, on film assembly, which allows rational access to films of specific composition and thickness (Fig. 1). A combination of UV-vis spectroscopy, ellipsometry, quartz-crystal microbalance (QCM), and atomic force microscopy (AFM) measurements are used to characterize film growth and to assess the effects of molecular charge, surface treatment, and cage structure on the deposition and growth of optically transparent thin films. Second, we leverage the intrinsic optical transparency to enable *in situ* spectroscopic characterization of post-synthetic metalation. Notably, while conventional MOF films assembled *via* established layer-by-layer methods rely on covalent or strong coordination interactions leading to extended crystalline frameworks,^8^ these structures often exhibit inherent grain boundaries, cracks, or surface defects which can reduce optical transparency due to increased light scattering. In contrast, the ionic self-assembly approach used here results in smoother, defect-free porous salt films characterized by fewer grain boundaries, as confirmed by AFM and ellipsometry measurements, thus yielding enhanced optical transparency essential for effective *in situ* spectroscopic characterization. By virtue of thickness control conveyed by layer-by-layer growth, we demonstrate depth dependence on metalation kinetics that is not readily apparent in bulk metalation experiments.

## Results and discussion

### Film deposition and growth

Our investigations of porous salt thin films were based on the simple hypothesis that layer-by-layer assembly would provide control over the thickness and composition of optically transparent thin films. The studies described here provide the rational basis for preparing films of specific thickness, which is prerequisite to application of porous thin films as platforms to investigate thickness-dependent reaction chemistry (*vide infra*).

Operationally, films are grown on a substrate surface, such as a glass slide, by alternating treatments with solutions containing salts of the target anion and cation, respectively. The growing film is briefly washed with fresh solvent between each exposure to prevent cross-contamination of the stock solutions and to remove physisorbed ions that are not coulombically incorporated into the growing film. The ionic (coulombic) interactions that guide our film formation inherently promote uniform layer deposition, resulting in homogeneous thin films without the grain boundaries or defects commonly observed in crystalline MOF films grown by traditional layer-by-layer approaches. SEM images of the porous salt films show even deposition of small particles on the substrate surface without cracks or obvious grain boundaries (Fig. S1). Complementary PXRD patterns revealed two broad diffraction peaks at 5.9° and 8.1° consistent with crystalline material and very small particle sizes (Fig. S2).

In the following discussion, a deposition cycle constitutes sequentially submerging the substrate in solutions of the anion and cation of interest. After each exposure to an electrolyte solution, the growing film is washed with fresh solvent. Film thickness was evaluated by complementary UV-vis spectroscopy and ellipsometry (Fig. S3); correlation of Beer's law behavior and film thickness was in alignment with previously reported ellipsometry experiments in related films.^32^ Additionally, QCM experiments confirm the linear relationship between deposition cycles and deposited mass (Fig. S4), which is in agreement with the optical and ellipsometry measurements.

Solutions of carboxylated porphyrins are prepared by treating the corresponding acids with triethylamine. We pair these anionic molecules with the triflate salt of a cationic zirconium-based coordination cage. We have previously shown that salts comprised of related ions are generally highly stable and can be prepared on various surfaces using facile deposition conditions.^32^ The anionic porphyrins were chosen for their tunable charge: dicarboxyphenylporphyrin, ([H_2_(dcpp)]^2−^) tetracarboxyphenylporphyrin, ([H_2_(tcpp)]^4−^) and octacarboxyphenylporphyrin ([H_2_(ocpp)]^8−^). Cationic zirconium-based cages were chosen for their chemical stability and availability of structural variation with the same overall charge: [Zr_12_(μ_3_-O)_4_(μ_2_-OH)_12_(Cp)_12_(*m*BDC)_6_]^4+^ ([Zr*m*BDC]^4+^), [Zr_12_(μ_3_-O)_4_(μ_2_-OH)_12_(Cp)_12_(Me_2_BDC)_6_]^4+^ ([ZrMe_2_BDC]^4+^), and [Zr_12_(μ_3_-O)_4_(μ_2_-OH)_12_(Cp)_12_(FDC)_6_]^4+^ ([ZrFDC]^4+^) (*m*BDC = 1,3-benzenedicarboxylate; Me_2_BDC = 2,5-dimethyl-1,4-benzenedicarboxylate; FDC = 2,5-furandicarboxylate).

Both triethylammonium salts of the included porphyrins and the triflate salts of the included Zr cages display excellent solubility in methanol. In contrast, the deposited porphyrin-cage salt is methanol insoluble while the remaining salt metathesis product, triethylammonium triflate, is methanol soluble. As such, deposition from methanolic solutions of the film components ensures that the porous salt is the surface-deposited phase without co-deposition of residual spectator triethylammonium or triflate.^32^

To utilize thin films as a platform to study solid-state chemical reactions, with specific interest in material growth and post-synthetic functionalization chemistry, we sought to clarify the mechanism and impact of synthesis variables on film growth. These studies are critical to systematic understanding the impact of molecular properties on the resultant assemblies.^33^

### Charge pairing as basis for deposition and growth

Work in our labs has shown that electrostatic interactions govern the bulk properties of porous salts where the solubility, stability, crystallinity, and processability of a given salt can be tuned by varying the charge of its constituent ions.^30^ The inherent charge of the constituent ionic components is vital in the synthesis of these solids; utilization of neutral starting cages and/or porphyrin does not lead to the formation of porous solids as the van der Waals interactions between these molecules presumably cannot overcome solvation of the molecular components.

**Fig. 2:**
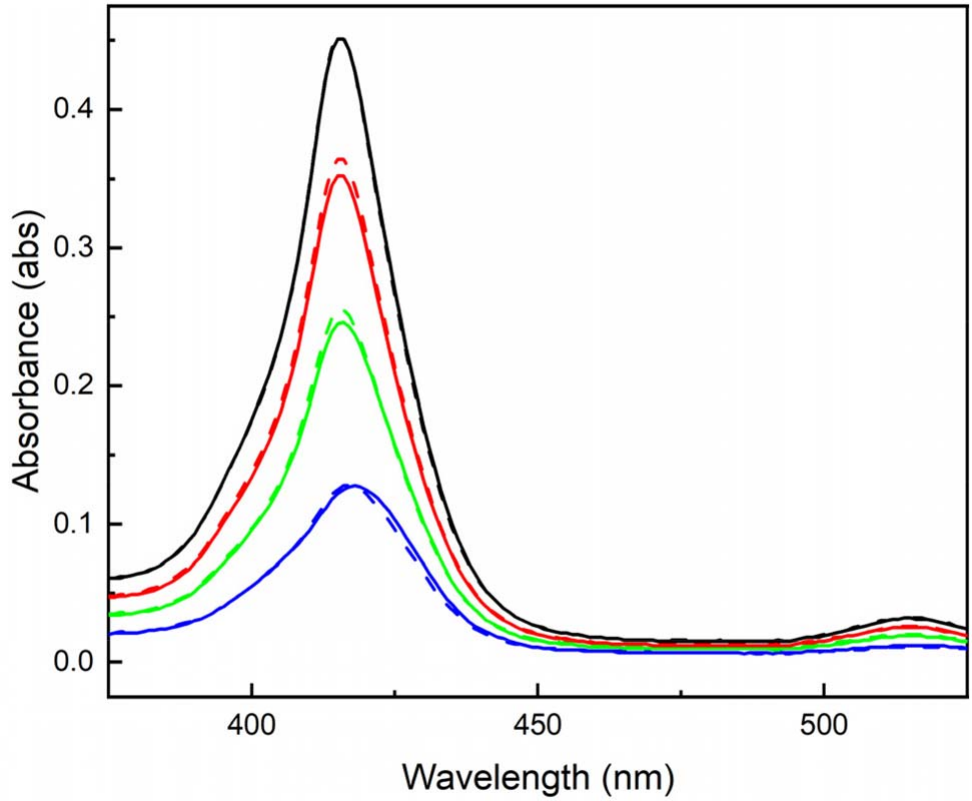
UV-vis absorption spectra of a [Zr*m*BDC][H_2_(tcpp)] thin film grown to 5 (blue), 10 (green), 15 (red), and 20 (black) deposition cycles. After every 5 cycles the film was exposed to the solution containing the target anion 10 additional times and a UV-vis spectra was collected (dashed line = additional 10 exposures).

To evaluate the hypothesis that layer-by-layer growth depends on chemisorption *via* coulombic pairing, and not physisorption, we evaluated the necessity for charge alternation on the growth of thin films. If cage/porphyrin interactions were not the dominant factor in solid deposition, we would expect subsequent exposures to either porphyrin or cage solutions alone would result in continued deposition of solid onto the surface of the film. To further test the role of electrostatic interaction in film deposition, we grew a 5-cycle film of [Zr*m*BDC]^4+^ and [H_2_(tcpp)]^4−^ as described above. After 5 deposition cycles, the film was further subjected to 10 additional exposures to the porphyrin solution only. As shown in Fig. 2, film growth is observed during deposition of the first 5 cycles, as evidenced by the growth of the porphyrin Soret band. In contrast, the subsequent 10 exposures to the porphyrin solution did not lead to an increase in absorbance. After thorough washing, the film was then resubjected to 5 additional deposition cycles and a large, nearly linear increase in absorbance was again observed while successive exposures to porphyrin solution alone did not deposit additional solid. Ultimately, exposure of the substrate to porphyrin solution does not lead to film growth if not mediated by cage exposures. These observations are consistent with charge pairing, and not physisorption of the film components, as responsible for film growth.

### Impact of substrate surface treatment

The surface chemistry of borosilicate glass, soda–lime glass, and quartz is complex and various charged species are accessible on each of these surfaces.^34^ Exposure of glass to alkaline conditions typically affords silanol groups that can be deprotonated to give surface-exposed –O anions that can serve as nucleation sites for the cationic cages employed in our films.^35,36^ We have previously shown that electrostatic approaches can be used to increase surface cage density on otherwise neutral surfaces.^32^ In the absence of some level of surface treatment, the density of such sites is low and variable.

**Fig. 3:**
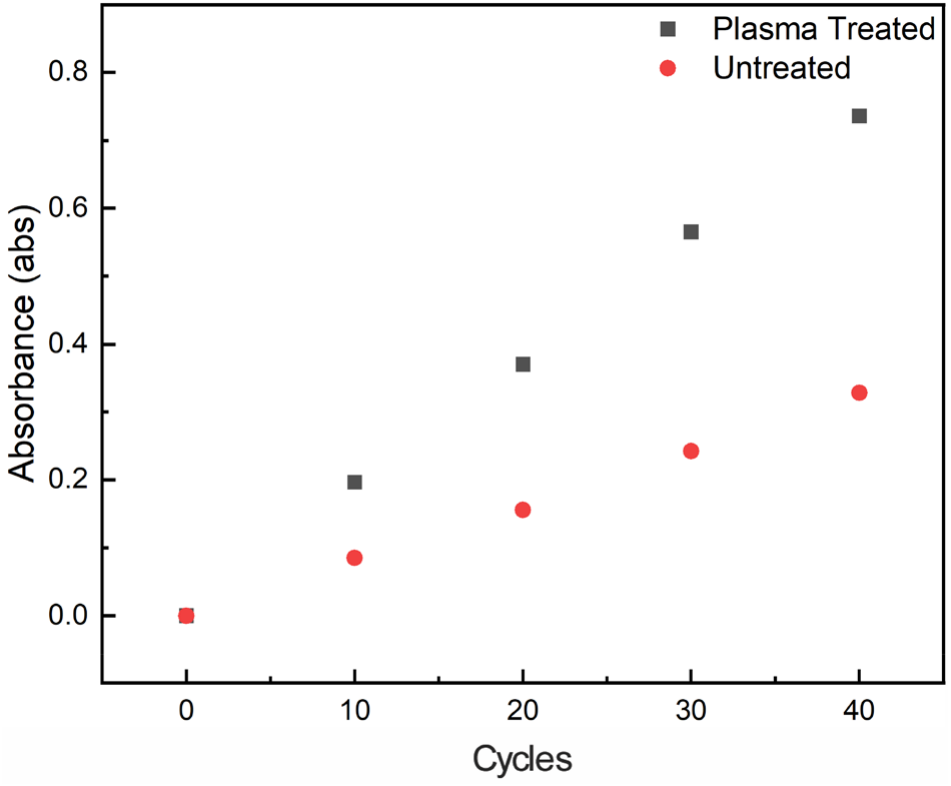
Plot of Soret band absorbance *versus* deposition cycles deposited for films grown on a plasma treated (black) glass slide as compared to an untreated (red) glass slide.

After confirming the impact of coulombic pairing on the growth of films (*vide supra*), we turned our attention to evaluating the impact of substrate surface charging on the deposition of films. To this end, we compared film deposition on untreated borosilicate glass, glass that had been plasma treated, and glass that had been surface silylated (*i.e.*, treated with trimethylsilyl chloride and triethylamine). Consistent with initial deposition *via* coulombic pairing, we observe significant changes in film growth for plasma-treated glass compared with untreated surfaces. After 10 deposition cycles a film grown on a treated surface shows a Soret band absorbance of 0.196 absorbance units while an untreated surface resulted in a film with an absorbance of 0.085 absorbance units. We observed a growth dependence consistent with increased initial deposition on the plasma-treated surface, in turn resulting in increased deposition in later cycles. This is consistent with the previously observed growth mechanism observed in related films which display a linear growth regime.^22^ Films grown on untreated surfaces displayed a linear deposition rate of 0.008 abs/cycle, whereas films grown on plasma treated glass slides showed improved deposition rates of 0.018 abs/cycle (Fig. 3). Similar growth rates were observed for films grown on quartz treated with a sodium hydroxide solution (Fig. S5). As a negative control, a glass slide was quickly immersed in a solution of trimethylsilyl chloride in dichloromethane, to silylate the glass-surface. Film growth on this slide showed half the absorbance at 10 deposition cycles compared to untreated glass (Fig. S6).

### Concentration dependence of film growth

**Fig. 4:**
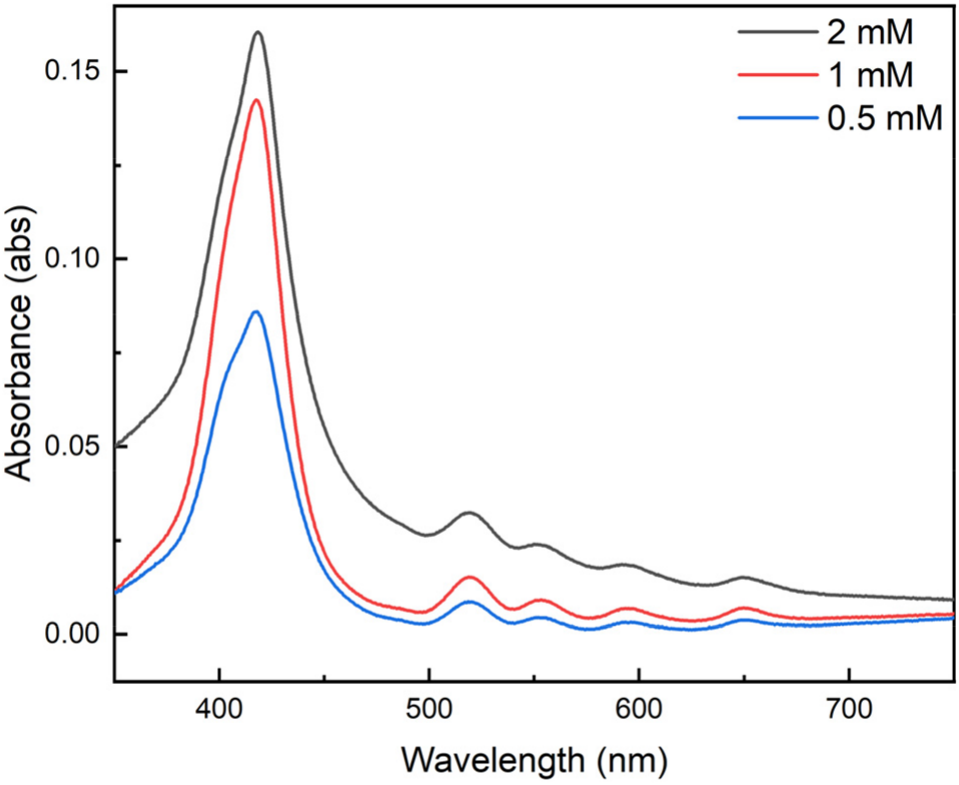
UV-vis spectra of optically transparent porous thin films grown to 5 deposition cycles using 0.5 mM solutions (blue), 1.0 mM solutions (red), and 2.0 mM solutions (black) of [Zr*m*BDC][OTf]_4_ and [HNEt_3_]_4_[H_2_(tcpp)] in methanol.

Growth of thin films comprised of [Zr*m*BDC]^4+^ and [H_2_(tcpp)]^4−^ is sensitive to the concentration of the ionic solutions from which films are grown (Fig. 4). Films grown to 5 cycles with 0.5 mM cage and porphyrin solutions displayed a characteristic Soret band absorbance of 0.086 absorbance units, while increasing the concentration of the solutions to 1.0 mM concentration resulted in nearly a doubling of film thickness as evidenced by the increase in Soret band absorbance to 0.142 absorbance units. Doubling the concentration of the cage and porphyrin solutions to 2.0 mM only slightly increased the film thickness/absorbance to 0.160 absorbance units while resulting in significant scattering as evidenced by the general rising baseline in absorbance across the entire spectral range. Beyond this, use of higher concentration solutions, particularly approaching the saturation limits of the cage or porphyrin, generally results in an increased rate of deposition. However, it is noted that saturated solutions afford lower-quality films as evidenced by rising baselines in UV-vis spectra indicative of bulk scattering associated with increased film opacity. Atomic force microscopy (AFM) images of films grown using concentrations below 1 mM displayed even film deposition across the substrate surface, further supporting our assertion that electrostatic self-assembly yields smooth, continuous surfaces free of notable cracks or defects (Fig. S7). To ensure sufficient deposition and minimize scattering, we used 0.5 mM concentration cation and anion solutions for all subsequent film preparations.

Exposure time has a minimal effect on film thickness for a given deposition cycle count (Fig. S8) and as such, a 5 seconds submersion in each solution was employed. The rise in absorbance per cycle is seen to be uniform, resulting in a linear growth regime. This linear growth regime is easily visualized by plotting the absorbance *versus* respective deposition cycle. To further corroborate the linear growth, films of 20, 40, 60, and 80 cycles were grown on separate pieces of glass, and their thickness was measured *via* ellipsometry (Fig. S3).^37^ The linear rise in thickness of films per deposition cycle further demonstrates the linear nature of growth of these films. Additionally, the mass of sample deposited in each cycle was evaluated using a quartz crystal microbalance and the resulting mass *vs.* deposition cycle plot (Fig. S4) validates the linear growth regime.

### Impact of molecular charge

As an additional means to control the growth and composition of porous salt films, the peripheral functionalization of the nonporous ion can be tuned to adjust charge, and thus the cage : porphyrin ratio and film thickness. Prior to this, our work in this regard has focused on [H_2_(tcpp)]^4−^-containing films given the relative ease of synthesis of this highly symmetric molecule and its ubiquity in porphyrin-containing MOFs.^38–40^ We have shown that this tetra-anionic species combines in a 1 : 1 ratio with tetra-cationic cages in both microcrystalline salts and thin films. To more fully investigate the role of porphyrin charge in film deposition, we prepared two additional porphyrin anions, 5,15-bis(4-carboxyphenyl)-10,20-diphenylporphyrin ([H_2_(dcpp)]^2−^) and meso-5,10,15,20-tetrakis-(3,5-dicarboxylatophenyl)porphyrin ([H_2_(ocpp)]^8−^) with 2^−^ and 8^−^ charges, respectively.

**Fig. 5:**
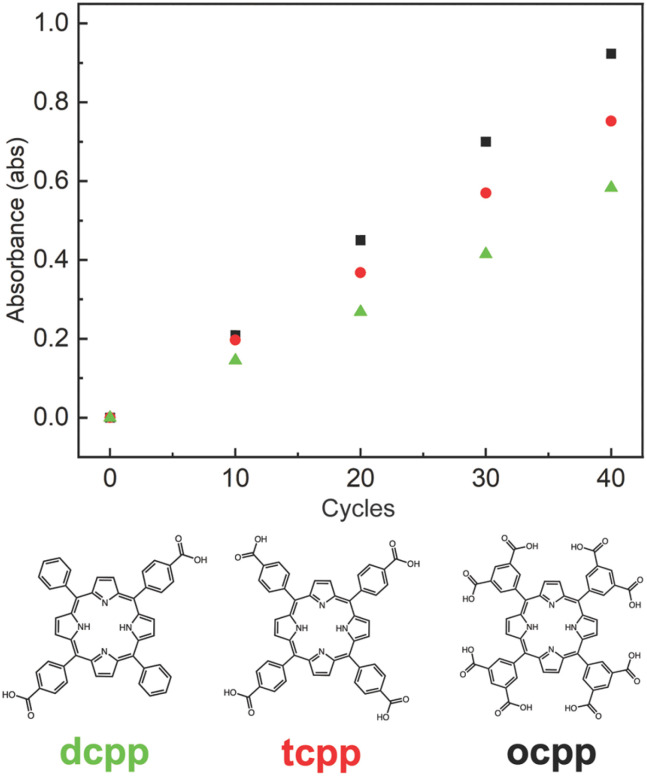
Deposition cycles *vs.* absorbance plots for films grown pairing [Zr*m*BDC]^4+^ with H_2_[H_2_(dcpp)] (green), H_4_[H_2_(tcpp)] (red), and H_8_[H_2_(ocpp)] (black).

Following our established film deposition protocol, we observe modest differences in rate of film deposition as a function of anion charge. In all three cases, linear growth is observed, as shown in the plot of absorbance *vs.* deposition cycle count (Fig. 5). As the three porphyrins have similar molar absorptivity, higher charge corelates with increased chromophore deposition per cycle. For a given formula unit of salt, it could be expected that larger quantities of cage would have to be deposited to balance the charge of higher charge anions where cage : porphyrin ratios of 1 : 2, 1 : 1, and 2 : 1 would be required for [Zr*m*BDC][H_2_(dcpp)]_2_, [Zr*m*BDC][H_2_(tcpp)], and [Zr*m*BDC]_2_[H_2_(ocpp)], respectively. The ratios of the respective salts were determined by ^1^H NMR of digested samples (Fig. S9–S11), revealing the cage : porphyrin ratio increases as the charge of the anion increases. However, in deposited films, we observe only a modest increase in deposited porphyrin for the three anions, suggesting the film deposition is limited by the available substrate surface area for porphyrin deposition and the associated cage deposition is governed by ion stoichiometry of the salts respectively. This is further supported by our thickness measurements where we observe similar thickness per deposition cycle for the different porphyrin molecules.

### Impact of cage structure

**Fig. 6:**
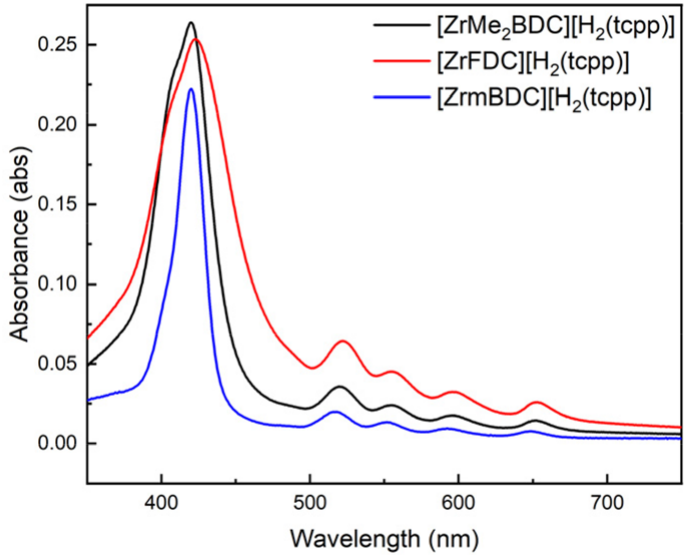
UV-vis spectra of 10 deposition cycle thin films comprised of [ZrMe_2_BDC][H_2_(tcpp)] (black), [ZrFDC][H_2_(tcpp)] (red), and [Zr*m*BDC][H_2_(tcpp)] (blue).

A unique benefit of porous salts is that the identity of the charged porous cage and the nonporous organometallic counterion can be independently varied. Zirconium-based cages offer advantages as the cationic components of porous salts as their size, geometry, charge, and functional groups can be independently modified.^29^ As we have previously shown that cage geometry does not play a significant role in salt porosity for porphyrin-based materials,^32^ we hypothesized that for a given cage charge, deposition and growth would not vary significantly. To assess this, we prepared two additional cages, ([ZrMe_2_BDC][OTf]_4_) and ([ZrFDC][OTf]_4_) and monitored the growth of free-base H_2_(tcpp) films using these structurally related species. As all three cages are tetra-cationic in nature, the extent of film deposition after 10 deposition cycle growth was largely independent of cage geometry (Fig. 6).

### Films as a platform to study post-synthetic metalation

The permanent porosity of the obtained thin films, confirmed by isothermal gas adsorption (Fig. S12), indicates a Langmuir surface area of 200 m^2^ g^−1^ for the [ZrmBDC][H_2_(tcpp)] film and provides a platform for post-synthetic metalation of the free-base porphyrin sites. Importantly, the optical transparency of the films allows *in situ* UV-vis spectroscopy to directly monitor metalation progress. The distinct spectral features of [H_2_(tcpp)]^4−^ and [Mn(tcpp)]^4−^ (Soret bands at 423 nm and 442 nm, respectively, in THF) make Mn(ii) an ideal probe for this transformation. Initial attempts using MnCl_2_ in methanol or THF—typical conditions for metalating porphyrin-containing MOFs^4,41,42^—were unsuccessful due to reversible ligation, oxidative side reactions, and the formation of spectrally ambiguous species (Fig. S13 and S14). To circumvent these issues, we employed Mn(HMDS)_2_ (HMDS = hexamethyldisilazide), which delivers both Mn(ii) and a strong, non-aqueous base to promote efficient metalation. This precursor provided clean and complete conversion to Mn(tcpp) without evidence of μ-oxo dimer [Mn(tcpp)]_2_O formation, a common byproduct in solution-phase reactions, especially in protic solvents. IR spectroscopy confirmed successful metalation (Fig. S15). Furthermore, the observation of complete metalation in thin films, along with the facile removal of residual Mn(HMDS)_2_ by simple washing, supports the persistence of permanent porosity throughout the metalation process. This conclusion is consistent with our prior work on related porous salt films, which also retained measurable surface areas and gas uptake following solid-state reactions.^32^

### Dependence of cage structure on metalation rate

With access to films comprised of three structurally related but geometrically diverse cages paired with the same [H_2_(tcpp)]^4−^ counterion, we were well positioned to investigate the impact of cage geometry on metalation rate. For these studies, we used films grown to 10 deposition cycles and comprised of [Zr*m*BDC][H_2_(tcpp)], [ZrMe_2_BDC][H_2_(tcpp)], or [ZrFDC][H_2_(tcpp)]. Upon treatment with Mn(HMDS)_2_ in THF under anerobic, anhydrous conditions, each of these films ultimately underwent complete metalation (*i.e.*, disappearance of the spectral features attributable to the [H_2_(tcpp)]^4−^ sites).

**Fig. 7:**
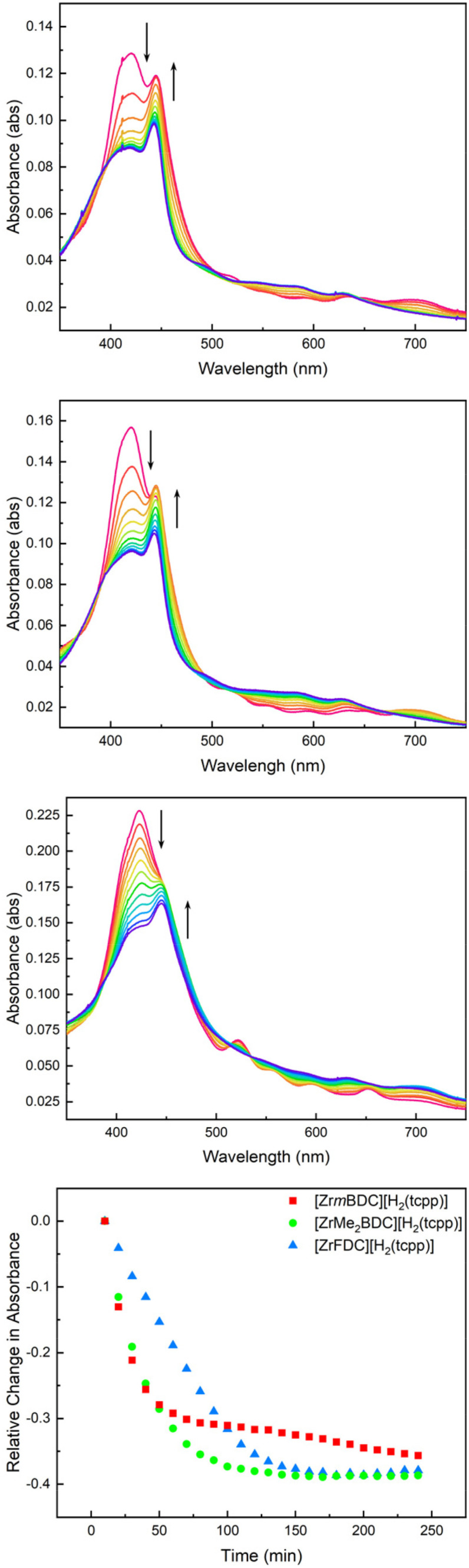
Time-dependent UV-vis spectra collected every 10 minutes during the metalation of 10 deposition cycle [Zr*m*BDC][H_2_(tcpp)] (top), [ZrMe_2_BDC][H_2_(tcpp)] (middle), and [ZrFDC][H_2_(tcpp)] (bottom) films with Mn(HMDS)_2_. The change in freebase Soret absorbance as a function of time illustrates a marked cage dependence of metalation.

Although extent of metalation is similar for the films based on different cages, we observed significant cage-dependent differences in metalation rate. Films comprised of [Zr*m*BDC]^4+^ displayed the fastest rates of metalation with the growth of the Mn(ii) Soret band at 442 nm and concomitant reduction of the freebase Soret band in less than 30 minutes (Fig. 7). Films grown using [ZrMe_2_BDC]^4+^ displayed a slower initial metalation rate and films grown with [ZrFDC]^4+^ displayed the slowest metalation rate (Fig. 7) with full metalation for these species observable after 120 and 180 minutes, respectively. We have previously shown that zirconium-based cages and their given salts have small but non-negligible differences in porosity,^19,20^ the pore size and shape of a given cage can have a large impact on the diffusion of molecules through the bulk film. This has been established for the adsorption of large gas molecules in cage-based porous solids.^43^

### Thickness dependence

As complete metalation was observed regardless of the identity of the cationic cage that was incorporated into a relatively thin film, we looked to increasing film thickness to assess the impact of sample dimensions on the extent and rate of metalation. In this way, the described thin films provide a unique opportunity in porous materials: crystalline samples are typically obtained as polycrystalline samples of different size and shape. Real time monitoring of the distribution of metalation sites is typically not possible so *ex situ* or indirect methods are required. By applying *in situ* spectroscopy, we can obtain distribution and kinetic data during post-synthetic metalation.

**Fig. 8:**
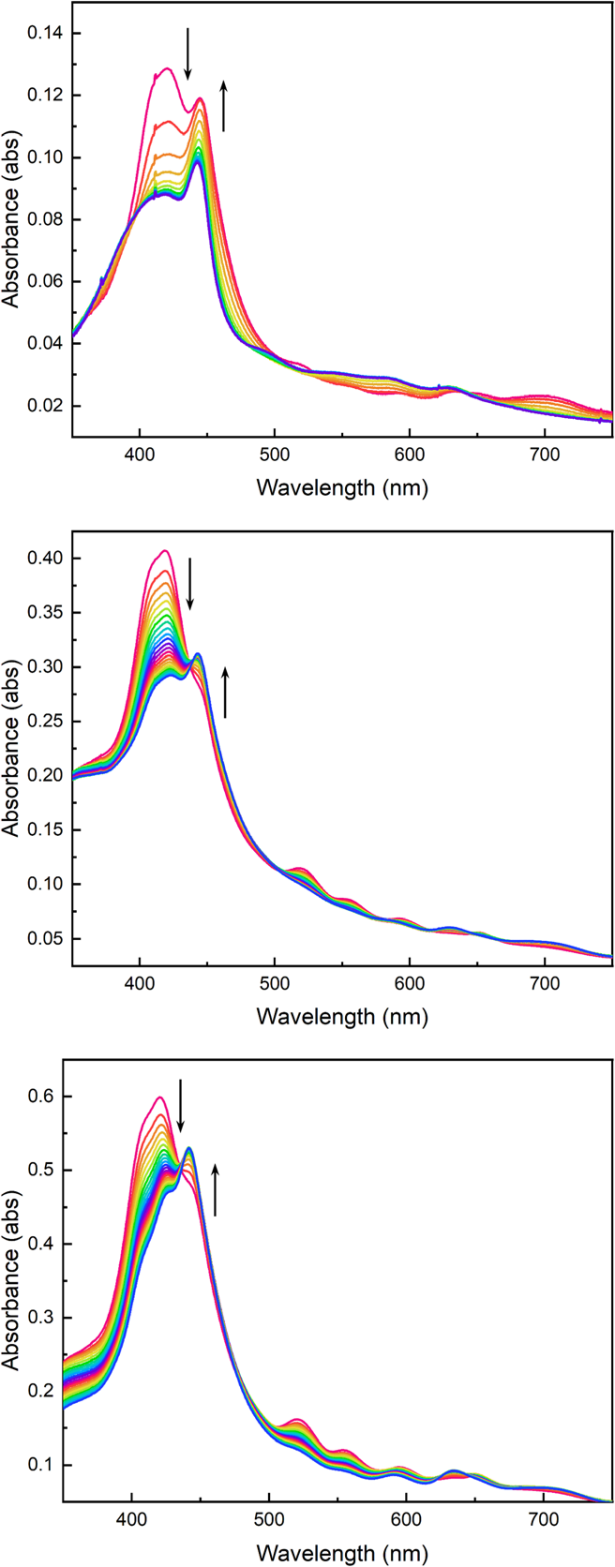
Time-dependent UV-vis spectra collected every 10 minutes during the metalation of 10 (top), 20 (middle), and 30 (bottom) deposition cycle [Zr*m*BDC][H_2_(tcpp)] films with Mn(HMDS)_2_.

**Fig. 9:**
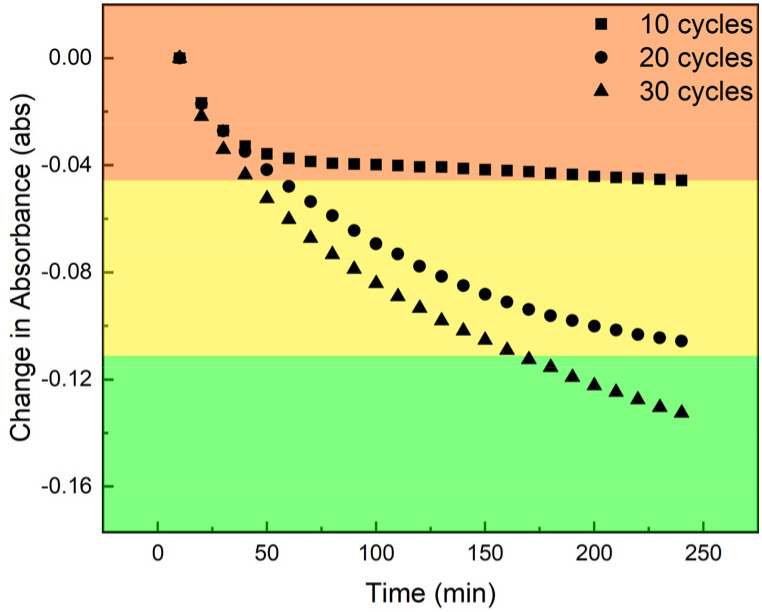
Change in freebase Soret band absorbance as a function of time illustrates the thickness dependence of metalation where the first ∼10 deposited layers of each film display rapid metalation while subsequent metalation is sluggish as a result of diffusion limitations in the thicker films.

We prepared films of [Zr*m*BDC][H_2_tcpp] using 10, 20, and 30 deposition cycles to afford films with maximum absorbances of 0.20, 0.44, and 0.66 at 423 nm, respectively. As noted above, the thinnest film displayed near complete metalation in approximately 60 minutes as evidenced by the evolution of Soret band position and intensity (Fig. 8) as well as the disappearance of the four Q-bands associated with freebase porphyrin and the appearance of two new Q-band absorbances (Fig. S16). In contrast, films grown to approximately double and triple the thickness, 20 and 30 cycles respectively, display nearly identical initial rates of metalation (Fig. 9) but are limited in their extent of metalation over reasonable timeframes. The thickest film that was investigated, based on 30 deposition cycles, showed non-negligible quantities of freebase porphyrin observable after even 8 hours of Mn(HMDS)_2_ exposure (Fig. S17 and S18). Global spectral fitting was done using a linear combination of the constituent free-base porphyrin and Mn(ii)-porphyrin spectra (details in SI). Analysis of these fits reveals that for all three films, initial metalation of what is equivalent to 10 deposited layers proceeds rapidly. More specifically the 10-cycle film, half of the 20-cycle film, and one third of the 30-cycle film were metalated in less than 30 minutes. Subsequent metalation of the remainder of the 20 and 30-cycle films was significantly slower with the latter still exhibiting a significant fraction of freebase porphyrin after 6 hours of Mn(HMDS)_2_ exposure.

These observations are consistent with diffusion-limited metalation. Though complete metalation may be possible for any thickness of material using this method, thicker films suffer from mass-transport issues making this technique most effective for thin films. This is analogous to reported core–shell modifications in metal–organic frameworks where diffusion issues limit the extent (*i.e.*, depth) of post-synthetic metalation.^44^ However, the high degree of tunability of molecular porous materials, coupled with the optical transparency of films based on these materials, offers significant advantages as the extent of metalation can be monitored throughout the film. The film thickness being easily tunable ameliorates diffusion limitations. This degree of characterization of *in situ* post synthetic metalation is not afforded to other porous materials due to their poor optical properties.

## Conclusions

The successful deposition and post-synthetic modification of optically transparent, porous salt thin films composed of carboxylated free-base porphyrin and cationic molecular cages underscore the value of these materials for probing the dynamics of functionalization of MOF-like materials. The permanent porosity and optical transparency of the films presented here facilitated *in situ* UV-vis spectroscopy, allowing real-time monitoring of the metalation process. Our results demonstrate that the rate and extent of Mn(ii) incorporation are dependent on both the structural characteristics of the cationic cage and the thickness of the film. Increasing film thickness revealed a significant thickness-dependence on metalation, with thicker films displaying slower and incomplete metalation within a given timeframe. These findings offer critical insights into the mechanisms governing post-synthetic modification in advanced porous solids, emphasizing the importance of film architecture in achieving desired material properties. The ability to precisely control film composition and thickness not only advances our understanding of porous solid functionalization but also lays the foundation for the design of advanced materials with tuneable catalytic and photocatalytic activities. The precise control over film architecture and composition demonstrated herein, coupled with real-time spectroscopic characterization, offers clear opportunities to investigate and optimize photocatalytic processes.

## Author contributions

This project was conceptualized by J. D. Simmons, S. Sarkar, D. C. Powers and E. D. Bloch with contributions from the collaborating authors. The film growth procedure was developed by J. D. Simmons, S. Sarkar, and A. Sur. Synthesis and characterization of the cage and porphyrin materials was performed by J. D. Simmons and S. Sarkar. The film thickness and roughness measurements were collected by A. A. Ezazi, S. Sarkar, and S. G. Fisher with supervision by J. C. Grunlan. The amide-based metal precursors were synthesized and characterized by A. D. Chivington and the *in situ* characterization of post-synthetic metalation was performed by J. D. Simmons. Independent reproducibility was demonstrated by M. N. Morey. The entire project was supervised by D. C. Powers and E. D. Bloch and manuscript was written by J. D. Simmons, S. Sarkar, D. C. Powers, and E. D. Bloch with contributions from the collaborating authors.

## Conflicts of interest

There are no conflicts to declare.

## Supplementary Material

SC-OLF-D4SC08061K-s001

## Data Availability

The data supporting this article have been included as part of the SI. The supplementary information includes detailed synthetic and experimental procedures as well as supporting UV-vis, IR, AFM, QCM, SEM, PXRD, NMR and isothermal gas adsorption data. See DOI: https://doi.org/10.1039/d4sc08061k.
