# Supplementary material for: In situ characterization of post-synthetic metalation in porous salt thin films
Source: Chem Sci. 2025 Jul 29;16(35):16284–92. doi: 10.1039/d4sc08061k (PMC12352658; doi:10.1039/d4sc08061k)
Supplement: SC-016-D4SC08061K-s001 [file SC-016-D4SC08061K-s001.pdf]

Supplementary Information (SI) for Chemical Science.
This journal is © The Royal Society of Chemistry 2025

Electronic Supplementary Information

In situ characterization of post-synthetic metalation in porous salt thin films

Joe D. Simmons,[a,‡] Subham Sarkar,[b,‡] Andrew A. Ezazi,[b] Aishanee Sur,[b] Ethan T. Iverson,[b] Merissa N. Morey,[a] Austin D. Chivington,[a] Sarah G. Fisher,[b] Jaime C. Grunlan,[b,c,d] David C. Powers,[b,*] and Eric D. Bloch[a,*]

[a]Department of Chemistry, Indiana University, Bloomington, Indiana 47405, United States

[b]Department of Chemistry, Texas A&M University, College Station, Texas 77843, United States

[c]Department of Mechanical Engineering, Texas A&M University, College Station, Texas 77843, United States

[d]Department of Material Science and Engineering, Texas A&M University, College Station, Texas 77843, United States

Email: powers@chem.tamu.edu, edbloch@iu.edu

Table of Contents

A. General Considerations S3
B. Synthesis and Characterization S4
C. Experimental Procedures S10
D. Film Growth Methodology S11
E. Additional Data S12
F. References S30

A. General Considerations

Materials Solvents were purchased as ACS reagent grade. Anhydrous solvents were taken from a Pure Process Technology solvent system and stored in a glovebox under an N_2 atmosphere over 3 angstrom or 4 angstrom sieves. Zirconocene dichloride, 2,5-furandicarboxylic acid, 2,5-dimethylbenzene-1,4-dicarboxylic acid, 1,3-benzenedicarboxylic acid, silver trifluoromethylsulfonate, triethylamine (NEt_3), 5,10,15,20-tetrakis(4-carboxyphenyl)porphyrin, manganese dichloride tetrahydrate, 2,6-lutidene, *N*,*N*-dimethylformamide (DMF), and methanol (MeOH) were purchased from Fisher Scientific. Glass slides were obtained from Environmental Monitoring Systems. Ethanol (EtOH) (KOPTEC 200 proof) was purchased from Decon Labs. Tetrahydrofuran (THF), diethyl ether (Et_2O), hexanes, ethyl acetate (EtOAc), dichloromethane (CH_2Cl_2), sodium borohydride, trifluoroacetic acid and acetic acid (AcOH) were obtained from Sigma Aldrich. Sulfuric acid (H_2SO_4) and hydrochloric acid (HCl) were obtained from VWR. 1H-Pyrrole, sodium bicarbonate ($NaHCO_3$), potassium hydroxide (KOH), triethylamine (NEt_3), and 2,3-dichloro-5,6-dicyano-1,4-benzoquinone (DDQ) were obtained from Oakwood Chemical. Propionic acid and 1,3,5-benzenetricarboxylic acid (trimesic acid) were obtained from Bean Town Chemical. 4-Carboxybenzaldehyde and ceric ammonium nitrate (CAN) were obtained from Alfa Aesar. Boron trifluoride diethyl etherate (BF_3•OEt) was obtained from Acros Organics. NMR solvents were purchased from Cambridge Isotope Laboratories and were used as received.

UV-vis Spectroscopy UV-vis spectra at Indiana University were obtained on a Jasco V750 UV-visible spectrophotometer. Continuous spectra were collected from 280-900 nm at a scan rate of 400 nm/min and a step size of 0.5 nm. UV-vis spectra collected at Texas A&M University were recorded on a Shimadzu 2501PC spectrometer with DH UV-vis-NIR light source from 250–800 nm at a scan rate of 500 nm/min and a step size of 0.5 nm. All spectra were collected using a reference of the solvent or solution in the sample cuvette.

Scanning Electron Microscopy Scanning electron microscopy images were obtained using an SEM/FIB Auriga 60 at Indiana University.

NMR ^{1}HNMR spectra were measured on a 500 MHz Bruker NMR spectrometer.

IR IR spectra were recorded on a Bruker VERTEX 70 at Texas A&M University, blanked against air, and were determined as the average of 32 scans.

Surface Treatment Glass slides were washed with distilled water, MeOH, and then distilled water once more. The surface of the slides was then plasma treated with an ATTO plasma cleaner (Diener Electronic, Ebhausen, Germany) for five minutes after being dried with filtered compressed air. Quartz cuvettes were washed with a 1M aqueous sodium hydroxide solution, distilled water, and then three times with MeOH prior to film growth.

Thickness Measurement Film thickness measurements were obtained using an Alpha-SE ellipsometer equipped with 623.8nm laser. QCM crystals were obtained from Inficon. The mass of deposited layers was measured using a Maxtek RQCM. Film growth procedure as follows - one minute soak time in cage solution, one minute rinse time in MeOH, air dry with compressed air, repeat soak in porphyrin, rinse in MeOH, air dry. Repeat full cycle 5x, equilibrate on RQCM for 10 minutes, measure mass. Wash solutions replenished every 10 cycles.

B. Synthesis and Characterization

Synthesis of $Zr_{12}(\mu_3\text{-}O)_4(\mu_2\text{-}OH)_{12}(Cp)_{12}(FDC)_6(OTf)_4$

This coordination cage was synthesized as previously reported.[1]

Synthesis of $Zr_{12}(\mu_3\text{-}O)_4(\mu_2\text{-}OH)_{12}(Cp)_{12}(Me_2BDC)_6(OTf)_4$

This coordination cage was synthesized as previously reported.[2]

Synthesis of $Zr_{12}(\mu_3\text{-}O)_4(\mu_2\text{-}OH)_{12}(Cp)_{12}(m\text{BDC})_6(OTf)_4$

This coordination cage was synthesized using a procedure adapted from the literature.[3]

Four 20 mL scintillation vials were each charged with zirconocene dichloride (0.292 g, 1.00 mmol), 1,3-benzenedicarboxylic acid (0.083 g, 0.50 mmol), DMF (17 mL), and deionized H_2O (0.5 mL). The reaction mixture was heated to 45 °C for 8 h. The obtained white crystalline solid was collected by centrifugation and the mother liquor was decanted. The resulting white solid was washed with chloroform five times over 3 d. The portions were combined in a 500 mL round bottom flask and dried under dynamic vacuum.

Following drying, the round bottom flask was wrapped in foil and a solution of silver triflate (0.306 g, 1.20 mmol) in MeOH (250 mL) was added. The reaction was stirred in darkness for 3 days. The resulting slurry was centrifuged, and the mother liquor was collected. The MeOH was removed under a dynamic vacuum resulting in a white solid. The MeOH solvated material was activated at 25 °C under dynamic vacuum to afford the title compound (1.06 g, 0.283 mmol) in 85% yield.

Synthesis of $[HNEt_3]_2$[5,15-bis(4-carboxyphenyl)-10,20-diphenylporphyrin]-($[HNEt_3]_2[H_2(dcpp)]$)

Synthesis of $[HNEt_3]_2[H_2(dcpp)]$ required the initial synthesis of 5-phenyl dipyrromethane, that was synthesized using an adapted literature procedure.[4]

Synthesis of 5-phenyl dipyrromethene (DMP)

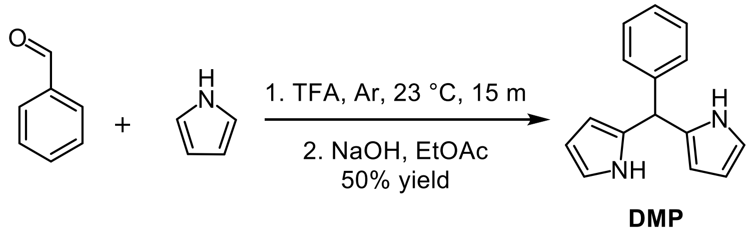

DMP

Pyrrole (5.600 mL, 8.020 mmol) and benzaldehyde (0.200 mL, 1.80 mmol) were added to a dry 50 mL round bottom flask and sparged with a stream of nitrogen for 10 min. Trifluoroacetic acid (0.016 mL, 0.20 mmol) was then added, and the solution was stirred under N_2 at room temperature for 15 min at which point no starting aldehyde was detected by TLC analysis. The mixture was diluted with CH_2Cl_2 (50 mL) and then washed with 0.1 M NaOH and then washed with water and dried over Na_2SO_4. The solvent was removed under reduced pressure. The resulting yellowish-brown solid was dissolved in minimum amount of eluant and purified by chromatography (80:20 Hexane/EtOAc). Any remaining pyrrole elutes first, followed slowly by dipyrromethene. The product fractions were collected and dried under dynamic vacuum yielding the title compound

(0.220 g) in a 50% yeild. ^1H NMR (δ, 23 °C, $CDCl_3$) δ 7.89 (bs, 2H, NH), 7.35–7.19 (m, 5H, ArH), 6.69 (q, 2H), 6.15 (q, 2H), 5.91 (m, 2H), 5.47 (s, 1H, meso- H).

Synthesis of [5,15-bis-(4-methoxycarbonylphenyl)-10,20-diphenylporphyrin] (H_2dcppOMe)

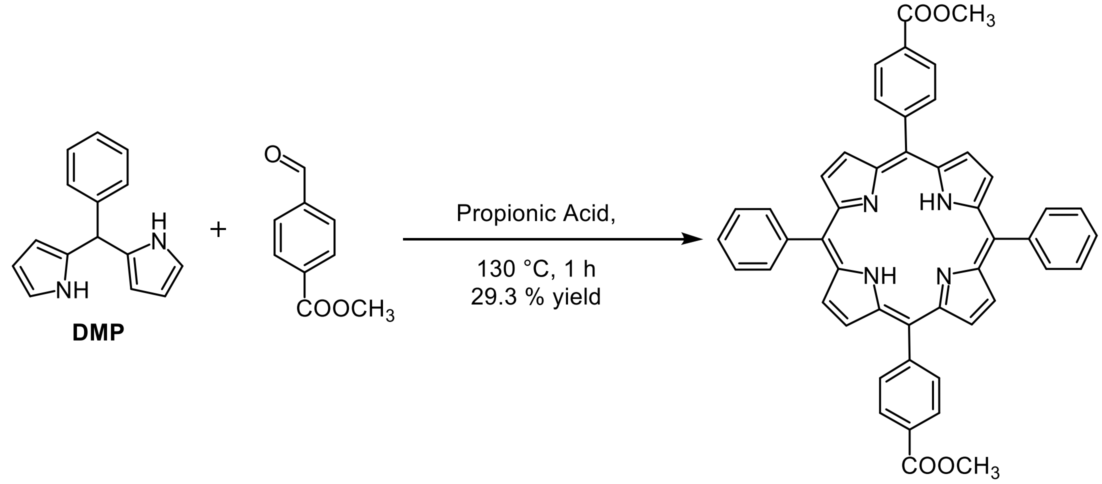

H_2dcppOMe was synthesized by using an adapted literature procedure.[5] A 100 mL round bottom flask was charged with DMP (0.450 g, 2.00 mmol) and methyl 4-formyl benzoate (0.164 g, 1.00 mmol) and propionic acid (100 mL) as solvent. The solution was rapidly stirred and refluxed for 1 h. The reaction mixture was allowed to settle, and the solvent was removed under reduced pressure. To the resulting black precipitate, 20 mL of EtOH was added. The solution was then filtered and washed with cold EtOH (3 × 15 mL). The resulting solid was dissolved in minimum amount of eluant and purified by flash column chromatography using CH_2Cl_2 as eluant. The fractions were collected, and the solvent removed under reduced pressure afforded the title compound as a purple solid (0.214 g) in a 29.3% yield.

Synthesis of $[HNEt_3]_2[H_2(dcpp)]$

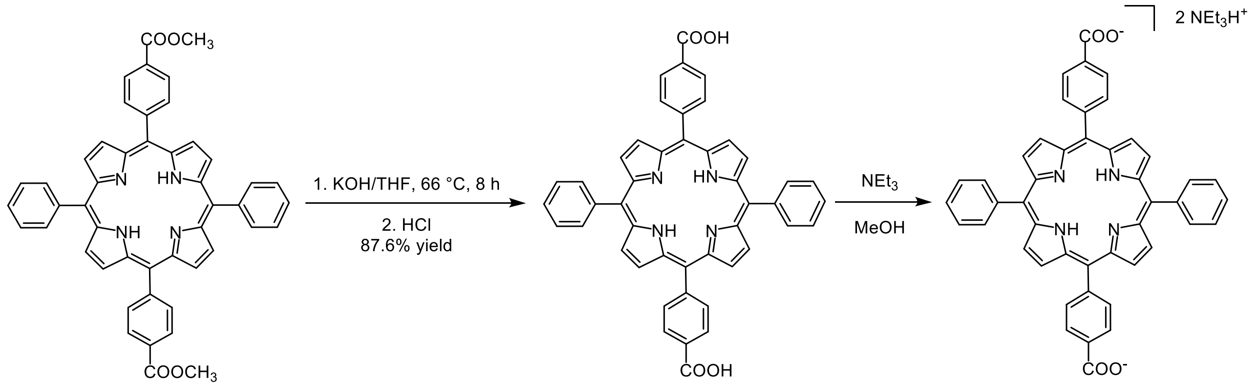

A 100 mL round bottom flask was charged with H_2dcppOMe (0.200 g, 0.270 mmol), THF (30 mL), and 2.00 mL of 0.1 M KOH. The solution was refluxed for 8 h and then quenched by adding 0.1 M HCl solution. The precipitate was collected by filtration and washed with deionized water (3 × 20 mL), to give the title purple compound (0.17 g) in a 87.6% yield. ^1H NMR (δ, 23 °C, DMSO-d_6): 13.32 (s, 2H), 8.85 (s, 8H), 8.37 (dd, J = 8 Hz, 8H), 8.23 (d, J = 4Hz, 4H), 7.85 (d, J = 4 Hz, 6H), –2.94 (s, 2H).

A 20 mL scintillation vial was charged with H_2(dcpp) (0.072 g, 0.10 mmol), MeOH (10 mL), and NEt_3 (0.014 mL, 1.0 mmol). The reaction mixture was stirred at 25 °C for 30 min, at which point all the solids dissolved to make a dark solution. The solution was dried under dynamic vacuum at 45 °C for 1 h to give a dark purple solid of $[HNEt_3]_4[H_2(dcpp)]$ (0.104 g, 0.094 mmol) in a 94% yield. ^{1}H NMR (δ, 23 °C, DMSO-d_6): 8.84 (s, 8H), 8.31 (m, 12H), 7.84 (d, J = 8 Hz, 8H), 2.63 (d, J = 8 Hz,12H), 1.01 (t, J = 4 Hz, 18H), –2.93 (s, 2H).

Synthesis of $[HNEt_3]_4$[tetrakis(4-carboxyphenyl)porphyrin]($[HNEt_3]_4[H_2tcpp]$)

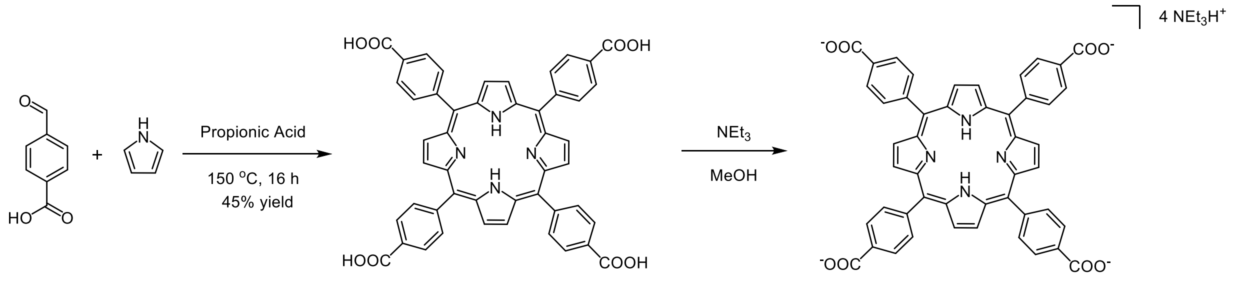

H_2tcpp was synthesized according to literature procedure.[6] A 250 mL round bottom flask was charged with 4-carboxybenzaldehyde (1.56 g, 10.3 mmol) and propionic acid (50 mL). The reaction mixture was heated at 80 ºC until the 4-carboxybenzaldehyde was completely dissolved. Freshly distilled 1H-pyrrole (0.70 mL, 0.010 mol) was added to the reaction solution and heated to 150 °C for 16 h. The reaction mixture was cooled to 23 °C and stored at –10 °C for several hours to induce precipitation. The precipitate was filtered and washed with CH_2Cl_2 (5 x 50 mL) and dried *in vacuo* to afford the H_2tcpp as a purple solid (0.89 g) in a 45% yield. ^{1}H NMR (δ, 23 °C, DMSO-d_6): 13.30 (s, 4H), 8.87 (s, 8H), 8.38 (q, J = 8 Hz, 16H), –2.94 (s, 2H).

A 20 mL scintillation vial was charged with H_2(tcpp) (0.079 g, 0.10 mmol), MeOH (10 mL), and NEt_3 (0.014 mL, 1.0 mmol). The reaction mixture was stirred at 25 °C for 30 min, at which point all the solids dissolved to make a dark solution. The solution was dried under dynamic vacuum at 45 °C for 1 h to give a dark solid of $[HNEt_3]_4[H_2(tcpp)]$ (0.107 g, 0.090 mmol) in a 90% yield. ^{1}H NMR (δ, 23 °C, DMSO-d$_6$): 8.87 (s, 8H), 8.37 (d, J = 8 Hz, 8H), 8.32 (d, J = 8 Hz, 8H), 2.63 (d, J = 8 Hz, 24H), 1.01 (t, J = 4 Hz, 36H), –2.91 (s, 2H).

Synthesis of $[HNEt_3]_8$[*meso*-5,10,15,20-tetrakis-(3,5-dicarboxylatophenyl)porphyrin] ($[HNEt_3]_8[H_2(ocpp)]$)

Synthesis of $[HNEt_3]_8[H_2(ocpp)]$ was done by using a procedure adapted from the literature.[7]

Synthesis of 1,3,5-benzenetricarboxylic acid triethylester

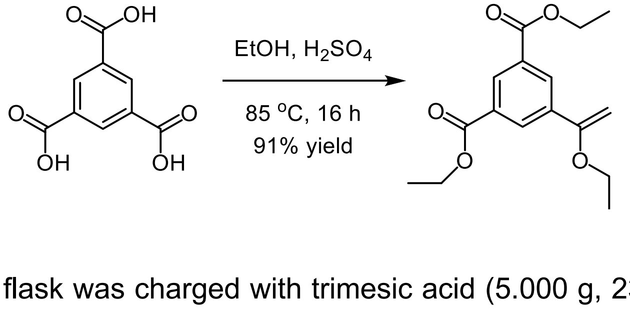

A 250 mL round bottom flask was charged with trimesic acid (5.000 g, 23.80 mmol), EtOH (125 mL), and H_2SO_4 (2 mL). The reaction mixture was heated at 85 °C for 16 h. The reaction mixture

was cooled to 23 °C, the observed white precipitate was isolated via vacuum filtration and washed with H_2O (1 x 100 mL) and EtOH (2 x 75 mL) and dried *in vacuo* to afford the title compound as a white solid (6.33 g) in a 91% yield. ^{1}H NMR (δ, 23 °C, DMSO-d_6): 8.65 (s, 3H), 4.40 (q, J = 8 Hz, 8H), 1.36 (t, J = 8 Hz, 12H).

Synthesis of Diethyl 5-Hydroxymethylisophthalate

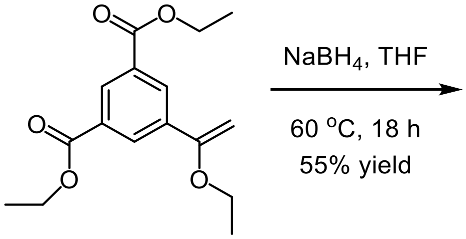

Under N_2, a 250 mL Schlenk flask was charged with 1,3,5-benzenetricarboxylic acid triethylester (4.640 g, 18.40 mmol), $NaBH_4$ (0.574 g, 15.1 mmol), and anhydrous THF (150 mL). The reaction mixture was heated to 60 °C for 18 h, during which time the solution turned red. The reaction mixture was cooled to –10 °C, and H_2O was added dropwise until bubbling ceased, after which 1 M HCl (10 mL) was added to give a clear solution. Solvent was removed *in vacuo* and the residue was partitioned between Et_2O (150 mL) and H_2O (100 mL). The organic phase was washed with brine (100 mL) and H_2O (100 mL). The organic phase was removed *in vacuo*, and EtOH (100 mL) was added to the resulting white residue. The obtained suspension was filtered via vacuum filtration to remove unreacted starting material. Solvent was removed *in vacuo* and the resulting residue was purified by SiO_2 gel column chromatography (2:3 EtOAc/Hexanes, R_f = 0.45) to give the title compound as a white solid (1.79 g) in a 55% yield. ^{1}H NMR (δ, 23 °C, $CDCl_3$): 8.59 (s, 1H), 8.22 (s, 2H), 4.81 (s, 2H), 4.41 (q, J = 8 Hz, 4H), 1.84 (br, 1 H), 1.41 (t, J = 8 Hz, 6H).

Synthesis of Diethyl 5-Formylisophthalate

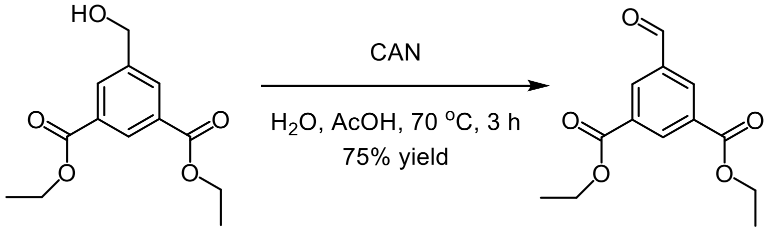

A 100 mL round bottom flask was charged with diethyl 5-hydroxymethylisophthalate (1.790 g, 7.100 mmol) and AcOH (20 mL). Ceric ammonium nitrate (10.000 g, 18.200 mmol) was dissolved in H_2O (20 mL) and added dropwise to the solution over 10 min. The reaction mixture was then heated to 70 °C for 3 h, during which the reaction mixture was observed to change color from red to yellow. The reaction mixture was cooled to 23 °C and partitioned between Et_2O (150 mL) and water (150 mL). The aqueous phase was extracted with Et_2O (2 x 100 mL). The combined organic layers were washed with saturated $NaHCO_3$ (1 x 100 mL) and brine (1 x 100 mL). Solvent was removed *in vacuo* to give the title compound as a white solid (1.34 g) in a 75% yield. ^{1}H NMR (δ, 23 °C, $CDCl_3$): 10.14 (s, 1H), 8.92 (s, 2H), 8.71 (s, 2H), 4.46 (q, J = 8 Hz, 4H), 1.44 (t, J = 8 Hz, 6H).

Synthesis of *meso*-5,10,15,20-Tetrakis-(3,5-diethoxycarbonylphenyl)porphyrin

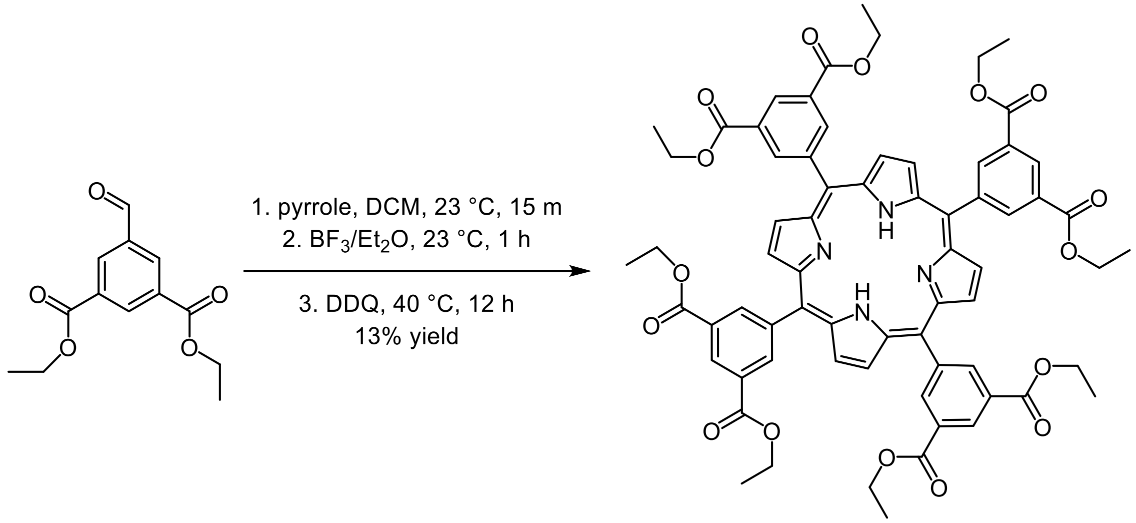

Under N_2, a 1 L three-necked round bottom flask was charged with diethyl 5-formylisophthalate (1.340 g, 5.350 mmol), freshly distilled 1H-pyrrole (0.360 mL, 5.30 mmol), and degassed CH_2Cl_2 (500 mL). The reaction mixture was stirred at 23 °C for 15 min, after which time BF_3•OEt (0.120 mL, 0.970 mmol) and the reaction vessel was moved to the dark. The reaction was stirred at 23 °C for 1 h, after which time DDQ (1.840 g, 8.110 mmol) was added, and the reaction was heated to 40 °C for 12 h. The reaction was cooled to 23 °C and solvent was removed *in vacuo*. The resulting residue was purified by SiO_2 gel column chromatography (19:1 CH_2Cl_2/EtOAc) to give a dark red solid. This solid was further purified by SiO_2 gel column chromatography (49:1 CH_2Cl_2/EtOAc) to give the title compound as a purple solid (0.41 g) in a 13% yield. ^1H NMR (δ, 23 °C, $CDCl_3$): 9.15 (s, 4H), 9.06 (s, 8H), 8.78 (s, 8H), 4.51 (q, J = 8 Hz, 16H), 1.43 (t, J = 8 Hz, 24H), –2.77 (br, 2H).

Synthesis of *meso*-5,10,15,20-Tetrakis-(3,5-dicarboxylatophenyl)porphyrin (ocpp)

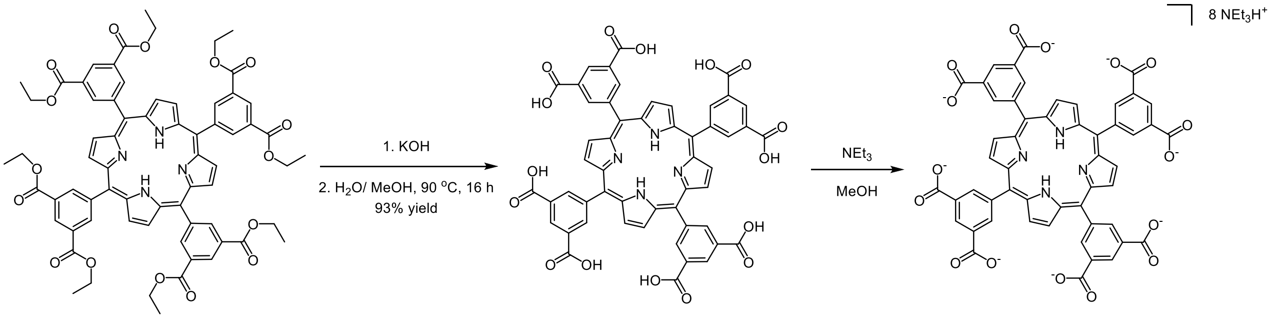

A 100 mL round bottom flask was charged with *meso*-5,10,15,20-Tetrakis-(3,5-diethoxycarbonylphenyl)porphyrin (0.400 g, 0.335 mmol), KOH (2.000 g, 3.570 mmol), H_2O (10 mL), and MeOH (60 mL). The reaction mixture was heated to 90 °C for 16 hours. The reaction mixture was cooled to 23 °C and acidified using 1 M HCl (10 mL). The observed precipitate was isolated via vacuum filtration and washed with water (2 × 30 mL) to yield H_2(ocpp) as a purple solid (0.302 g) in a 93% yield.

A 20 mL scintillation vial was charged with H_2(ocpp) (0.096 g, 0.100 mmol), MeOH (10 mL), and NEt_3 (0.014 μL, 1.000 mmol). The reaction mixture was stirred at 25 °C for 30 min, at which point

all the solids dissolved to make a dark solution. The solution was dried under dynamic vacuum at 45 °C for 1 hour to give a dark purple solid of $[HNEt_3]_4[H_2(ocpp)]$ (0.126 g, 0.092 mmol) in a 93% yield. ^1H NMR (δ, 23 °C, DMSO-d_6): 8.93 (s, 4H), 8.83 (m, 16H), 2.77 (d, J = 8 Hz, 48H), 1.07 (t, J = 4 Hz, 72H), –2.89 (s, 2H).

Synthesis of Bis[bis(trimethylsilyl)amino]manganese(II).

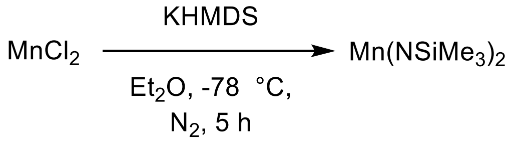

Synthesis of $Mn(HMDS)_2$ was done by using an adaptation of literature procedure.[8] Under rigorous exclusion of oxygen and moisture, a Schlenk flask was charged with anhydrous $MnCl_2$ (1.600 g, 12.00 mmol) and 50 mL of Et_2O. The flask was cooled in a cold well for 15 min with dry ice. Potassium bis(trimethylsilyl)amide (5.080 g, 25.40 mmol) was added to the stirring $MnCl_2$ slurry slowly over the course of 5 min. The mixture was then stirred at room temperature for 5 h. At this point it was observed all the ether insoluble $MnCl_2$ had been taken into solution and a fine white precipitate was observed in the light brown solution. The mixture was then dried, redissolved in hexanes and filtered through a pad of Celite to remove precipitated salts. Pale crystals of bis[bis(trimethylsilyl)amino] manganese(II) [$Mn(HMDS)_2$] were collected by cooling a concentrated pentane solution giving multiple crops.

C. Experimental Procedures

Post synthetic metalation of films

Freebase porphyrin films of varying thicknesses were grown on the inside of a screw cap quartz cuvette. The films were desolvated at room temperature under dynamic vacuum for 12 hours prior to metalation. A solution of $Mn(HMDS)_2$ (0.015 g, 0.040 mmol) in 8 mL of anhydrous THF, was split between the cuvette containing the film and the reference cell. The cuvettes were capped and sealed with Teflon tape and the reaction was monitored by UV-vis spectroscopy.

Silylation of glassware

Borosilicate glass slide was cut into 1 cm by 3 cm pieces. Under an N_2 atmosphere, the glass slide was added to a 20 mL scintillation vial containing 5% trimethylsilyl chloride in anhydrous toluene. The slide was left in the solution for 30 min. Following this, the slide was washed with anhydrous toluene and anhydrous MeOH. It was then allowed to dry, brought out to air, and put in a >100 ˚C oven and left overnight.

Measurement of film thickness

Borosilicate glass slides (42 mm x 9 mm) were washed with MeOH and water to remove any unwanted particles, and air-dried. Films of 5, 10, 15, and 20 deposition cycles were GROWN on 4 different glass slides using standard procedure and the slides were washed with fresh MeOH prior to activation under dynamic vacuum at room temperature. Following this, the thickness of the films was measured using an Alpha-SE ellipsometer equipped with 623.8nm laser.

Fitting methods for kinetics of film metalation

A 20 cycle [Zr*m*BDC][Mn(tcpp)] film was prepared by photoreduction of a [Zr*m*BDC][Mn(tcpp)Cl] film as previously reported,[9] the spectral features of this Mn(II) film were recorded. 10, 20, 30 cycle films of [Zr*m*BDC][H_2(tcpp)] were prepared, and their UV-vis spectra were recorded. Fitting of the metalation of 10, 20, 30 cycle films was done in Origin, using a linear combination function: AB = (C1*A + C2*B)*C3; where,

AB = combined spectrum representative of metalation
A = Spectrum of [Zr*m*BDC][H_2(tcpp)]
B= Spectrum of [Zr*m*BDC][Mn(tcpp)]
C1, C2 = parameters for contributions of A, B respectively
($0 \leq C1 \leq 1$, $0 \leq C2 \leq 1$, and C2= 1 – C1.)

C3 = variable for intensity of fitting

D. Film Growth Methodology

Growing films in quartz cuvettes: Films were grown using equimolar 0.5 mM MeOH solutions of a cationic cage triflate salt and a triethylammonium porphyrin salt. A quartz cuvette was washed with an aqueous 1 M solution of sodium hydroxide; followed by 3 washes with deionized water and 3 washes with MeOH. The cage and porphyrin solutions were alternately dispensed into the quartz cuvette and then removed mediated by washes with fresh anhydrous MeOH. The solutions were kept in the cuvette for 5 seconds and the MeOH washes were slightly longer at 5-10 seconds. Care was taken to ensure the ion solution was entirely removed from the cuvette prior to washing to minimize cross contamination as this leads to precipitation and poor film quality. The films were washed 3 additional times with MeOH prior to activation under dynamic vacuum at room temperature.

Growing films on glass slide: Films were grown using equimolar 0.5 mM MeOH solutions of a cationic cage triflate salt (solution 1) and triethylammonium porphyrin salt (solution 2). A plasma-treated glass slide (42 mm × 9 mm) was dipped in a solution 1 for 5 min. The slide was then dipped in fresh MeOH for 10 seconds. The washed slide was then dipped in solution 2 for 5 seconds. The slide was dipped in MeOH for 10 seconds. Subsequent deposition cycles were made by sequential dips in the cage solution 1 (5 seconds), MeOH (10 seconds), porphyrin solution 2 (5 seconds), MeOH (10 seconds). After each 10 cycles, the wash solutions were replaced with fresh MeOH. The deposition process was repeated until the desired thickness was achieved. The slides were washed with fresh MeOH prior to activation under dynamic vacuum at room temperature.

Growing films on glass beads: Films were grown using equimolar 0.5 mM MeOH solutions of a cationic cage triflate salt and triethylammonium porphyrin salt. Glass beads were washed with an aqueous 1 M solution of sodium hydroxide; followed by 3 washes with deionized water and 3 washes with MeOH. Mediated by washes with fresh anhydrous MeOH, the cage and porphyrin solutions were alternately dispensed into a glass fritted funnel containing the glass beads and subsequently removed using vacuum filtration. The beads were kept in the solutions for 5 seconds and the MeOH washes were slightly longer at 5-10 seconds. Films were grown using 70 cycles and then washed with MeOH until the eluent was colorless. The films were transferred to a gas adsorption sample tube and activated under dynamic vacuum for 48 hours.

E. Additional Data

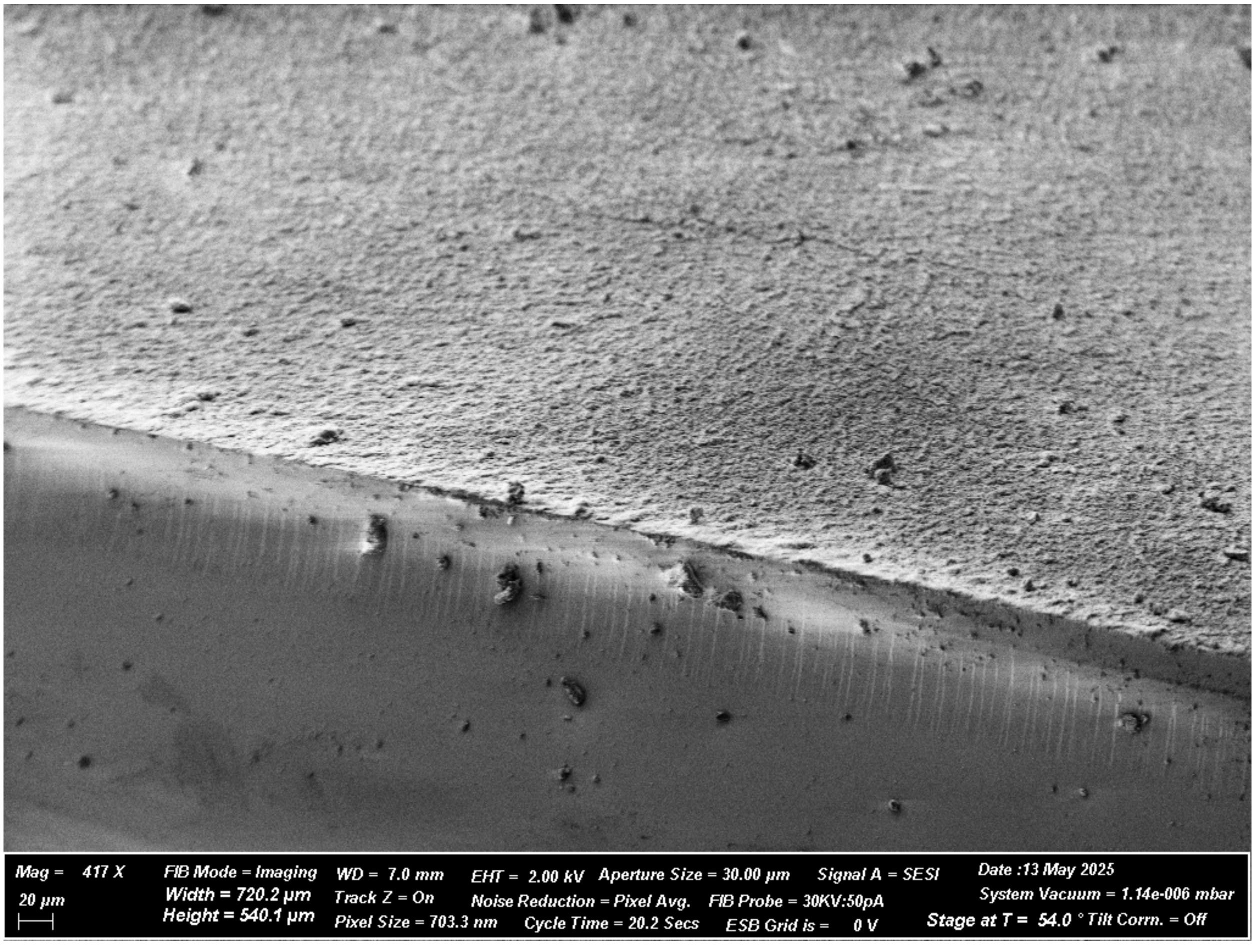

Figure S1: SEM images of a [Zr*m*BDC][H_2(tcpp)] film grown on glass.

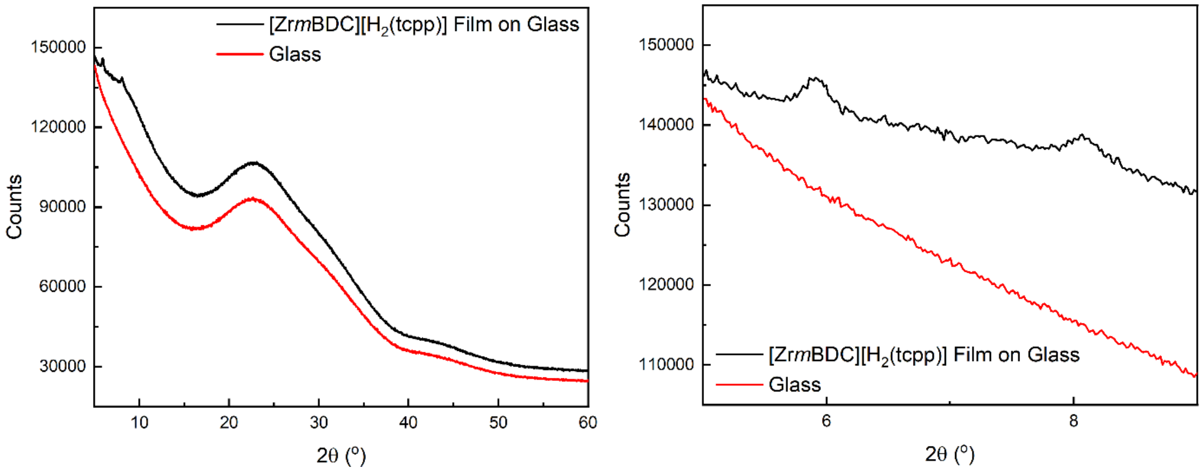

Figure S2: PXRD pattern of a [Zr*m*BDC][H_2(tcpp)] film grown to 30 deposition cycles on glass vs glass alone. Full pattern (Left) and small angles (Right).

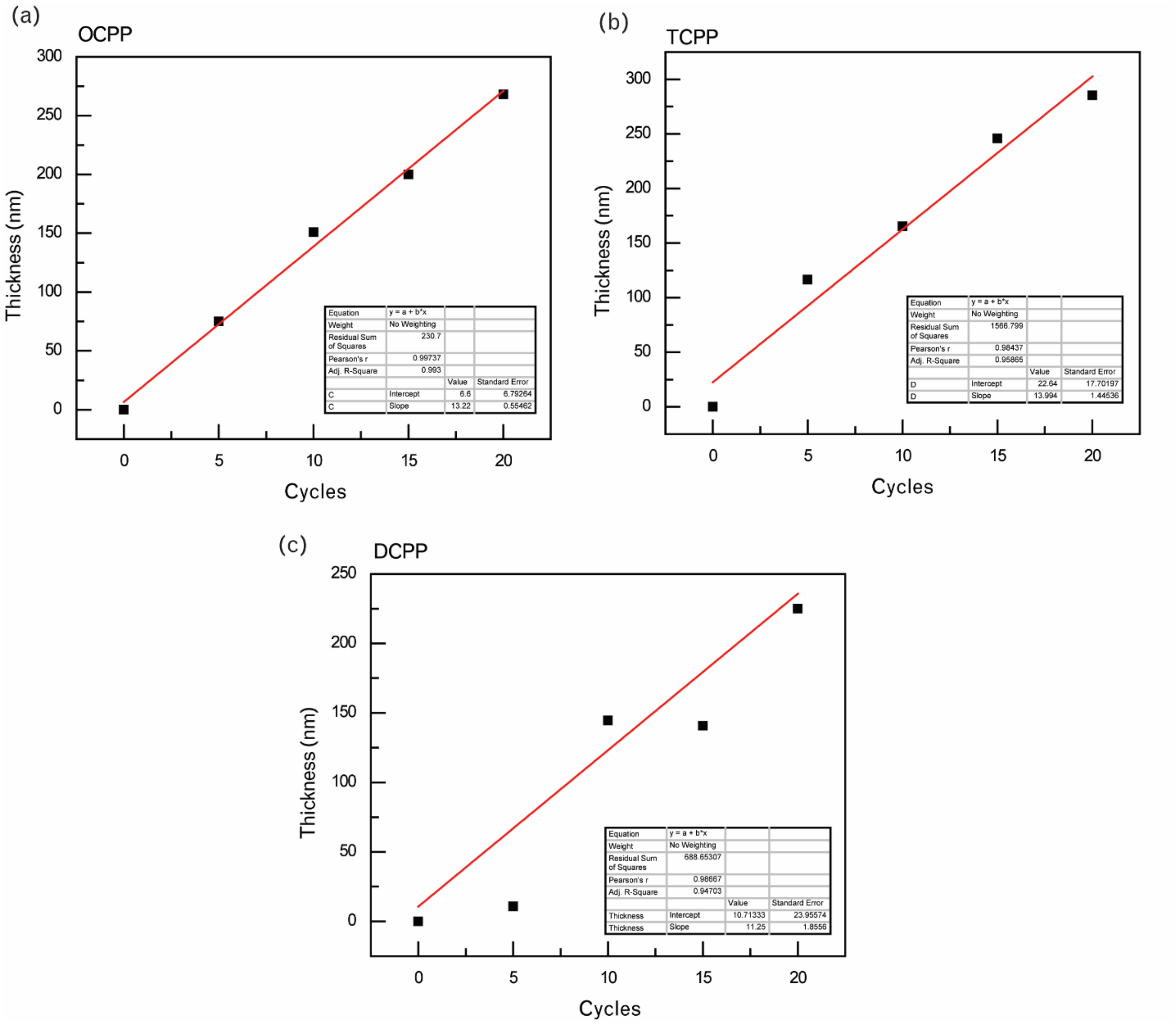

Figure S3: Plot of deposition cycles count vs. thickness (obtained by ellipsometry measurements) for the growth of thin films of (a) [Zr*m*BDC]$_2$[H$_2$(ocpp)], (b) [Zr*m*BDC][H$_2$(tcpp)], and (c) [Zr*m*BDC][H$_2$(dcpp)]$_2$ respectively.

(a) (b)

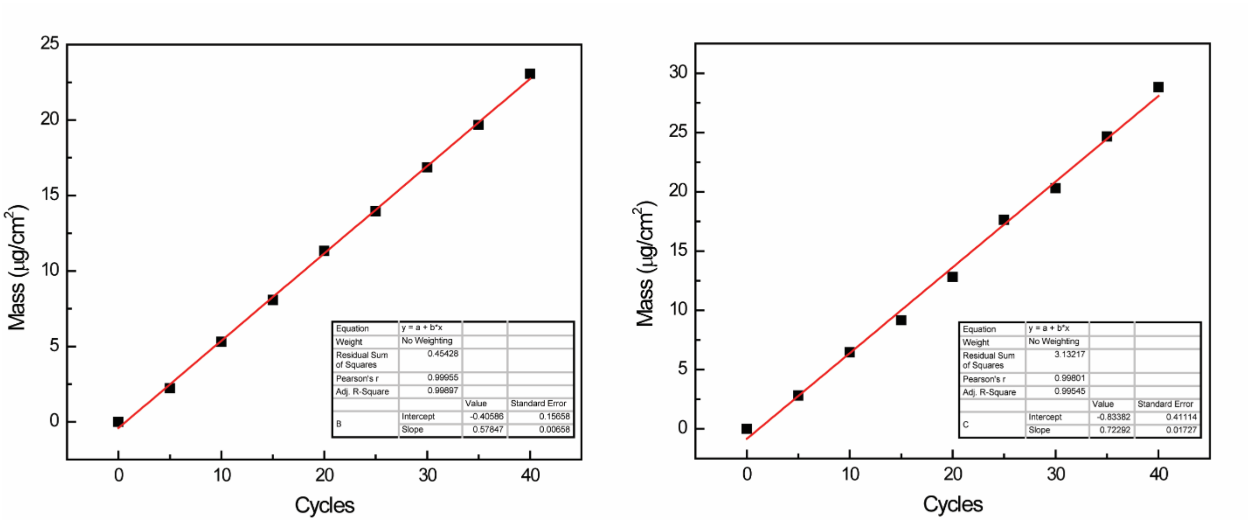

Figure S4: Quartz crystal microbalance (QCM) measurements for [Zr*m*BDC][H_2tcpp] films grown using solutions of (a) 0.5 mM and (b) 0.08 mM.

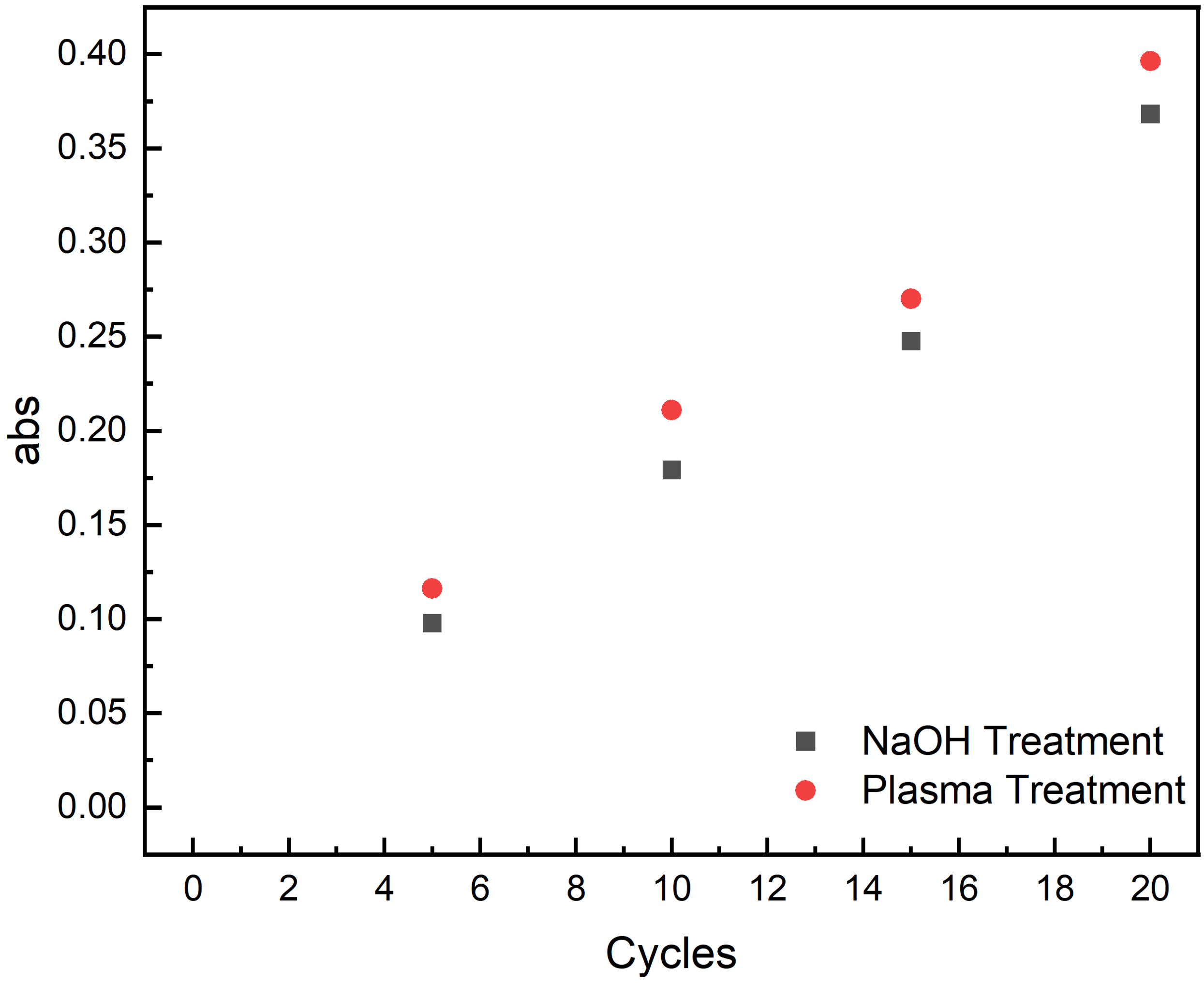

Figure S5: Comparison of [Zr*m*BDC][H_2(tcpp)] films growth on glass slides treated with NaOH or plasma charging.

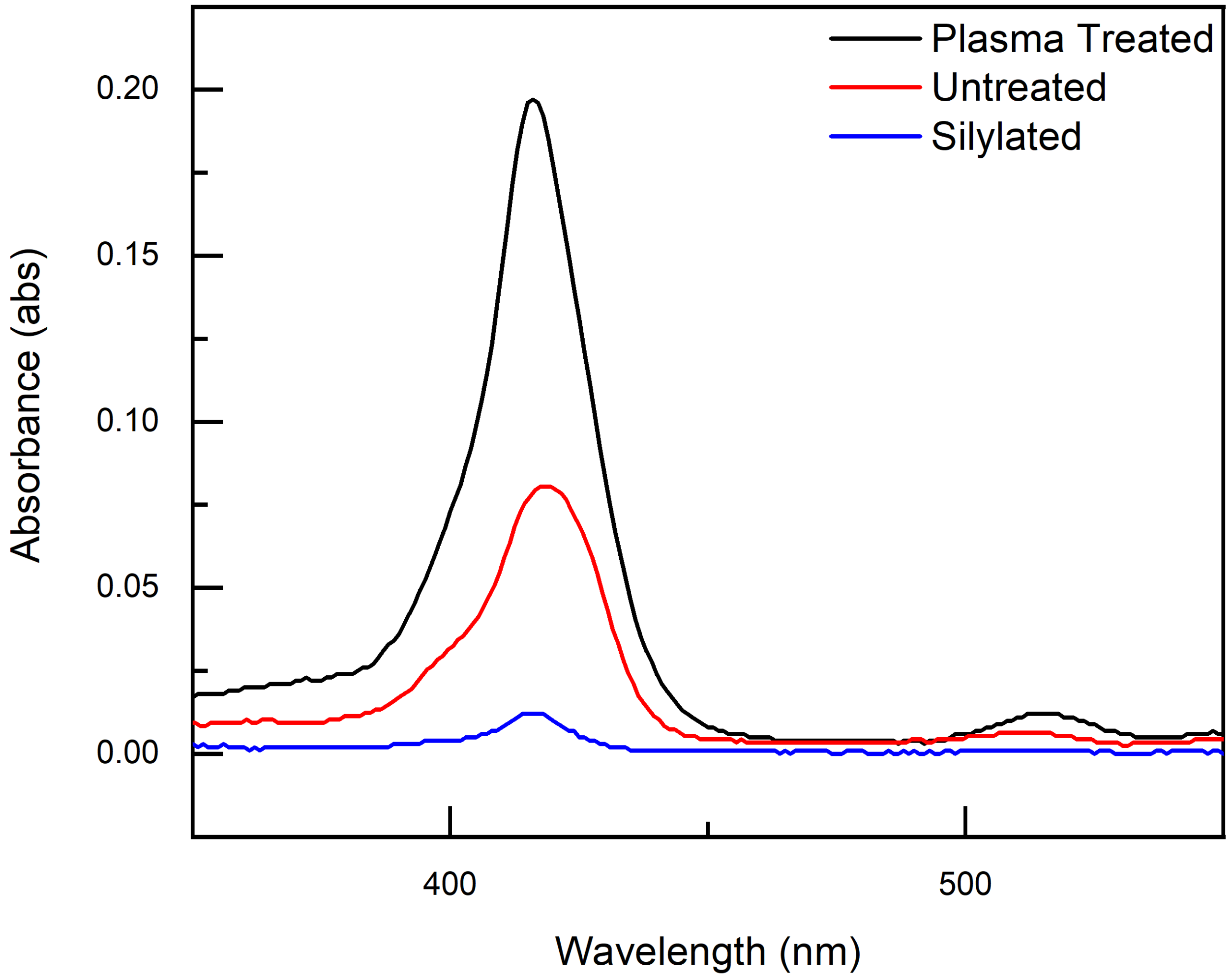

Figure S6: Comparison of 10 cycle [Zr*m*BDC][H_2(tcpp)] films growth on plasma treated, untreated, and silylated glass slides.

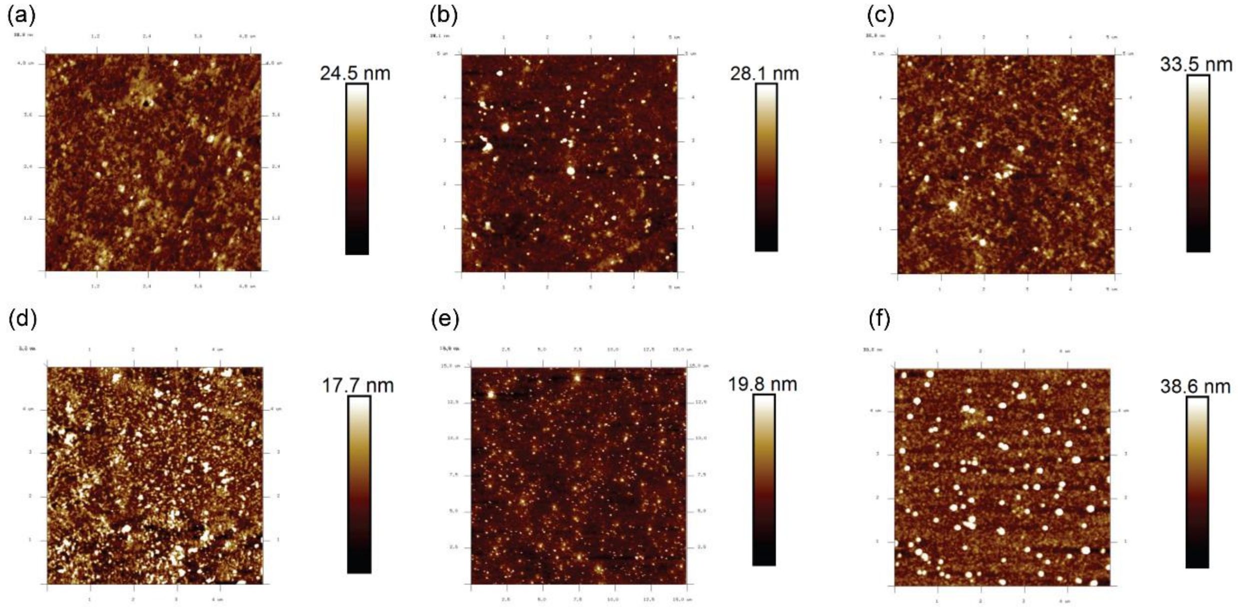

Figure S7: AFM images of [Zr*m*BDC][H_2tcpp] films grown at varied concentration. All images taken are 5 x 5 µm. (a) 1 mg/mL 10 cycles. (b) 1 mg/mL 20 cycles. (c) 1 mg/mL 30 cycles. (d) 0.25 mg/mL 5 cycles. (e) 0.25 mg/mL 20 cycles. (f) 0.25 mg/mL 30 cycles.

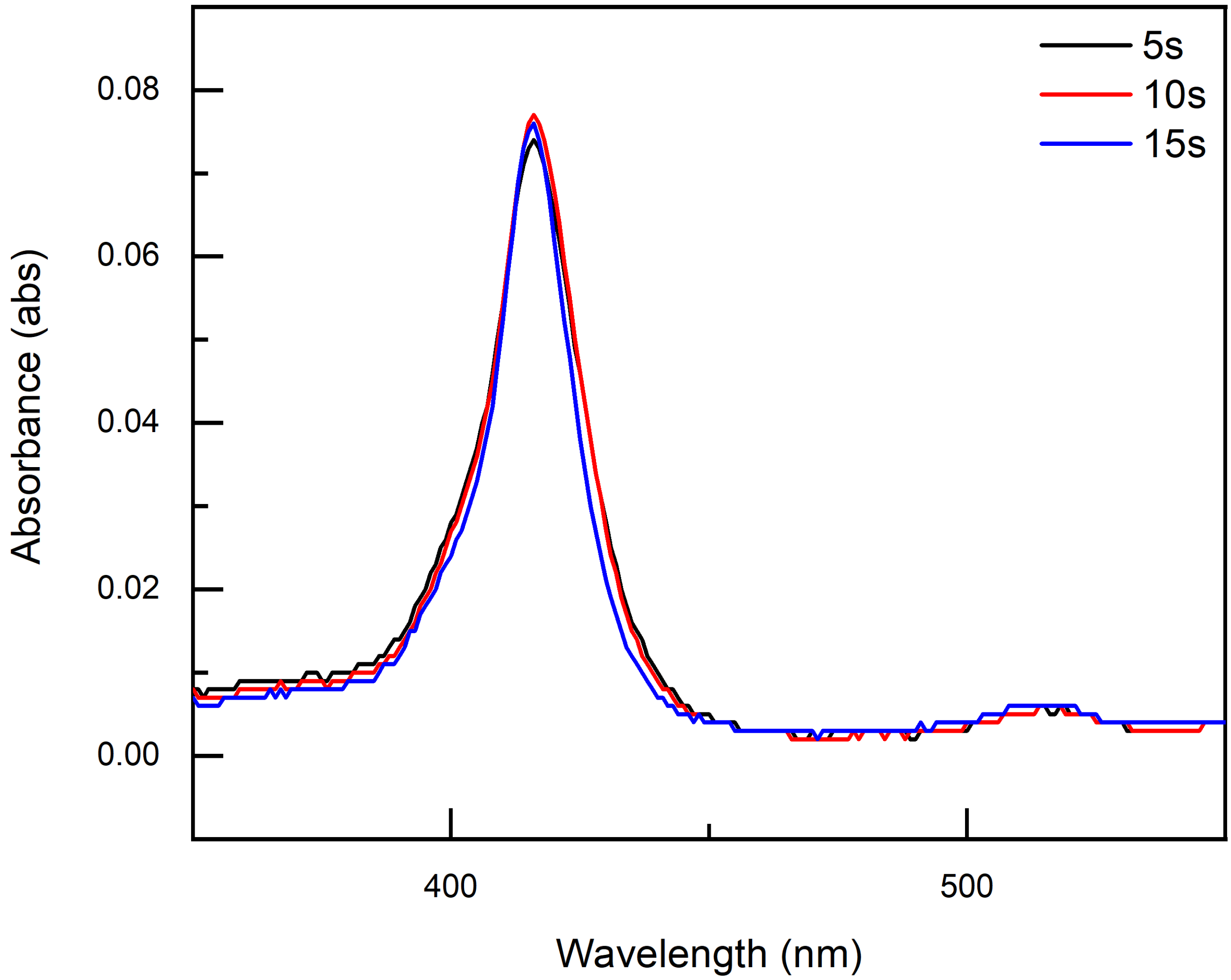

Figure S8: Plot of 5 cycle [Zr*m*BDC][H_2(tcpp)] films grown using 5, 10, and 15 second exposures to the target ion solutions.

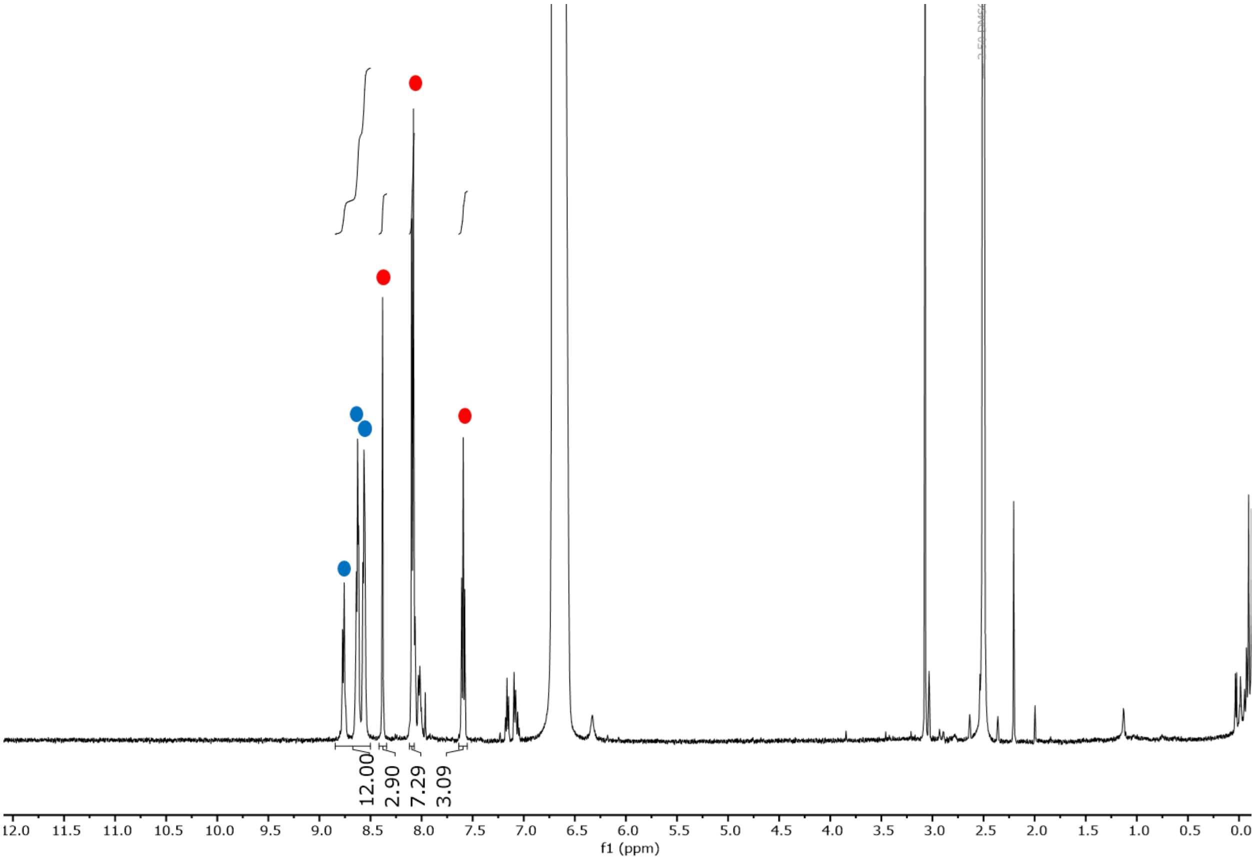

Figure S9: ^{1}HNMR spectra of [Zr*m*BDC][H_2dcpp]$_2$ digested with 5 drops of DCl in d_6-DMSO. Porphyrin peaks are labeled in blue and cage peaks are labeled in red.

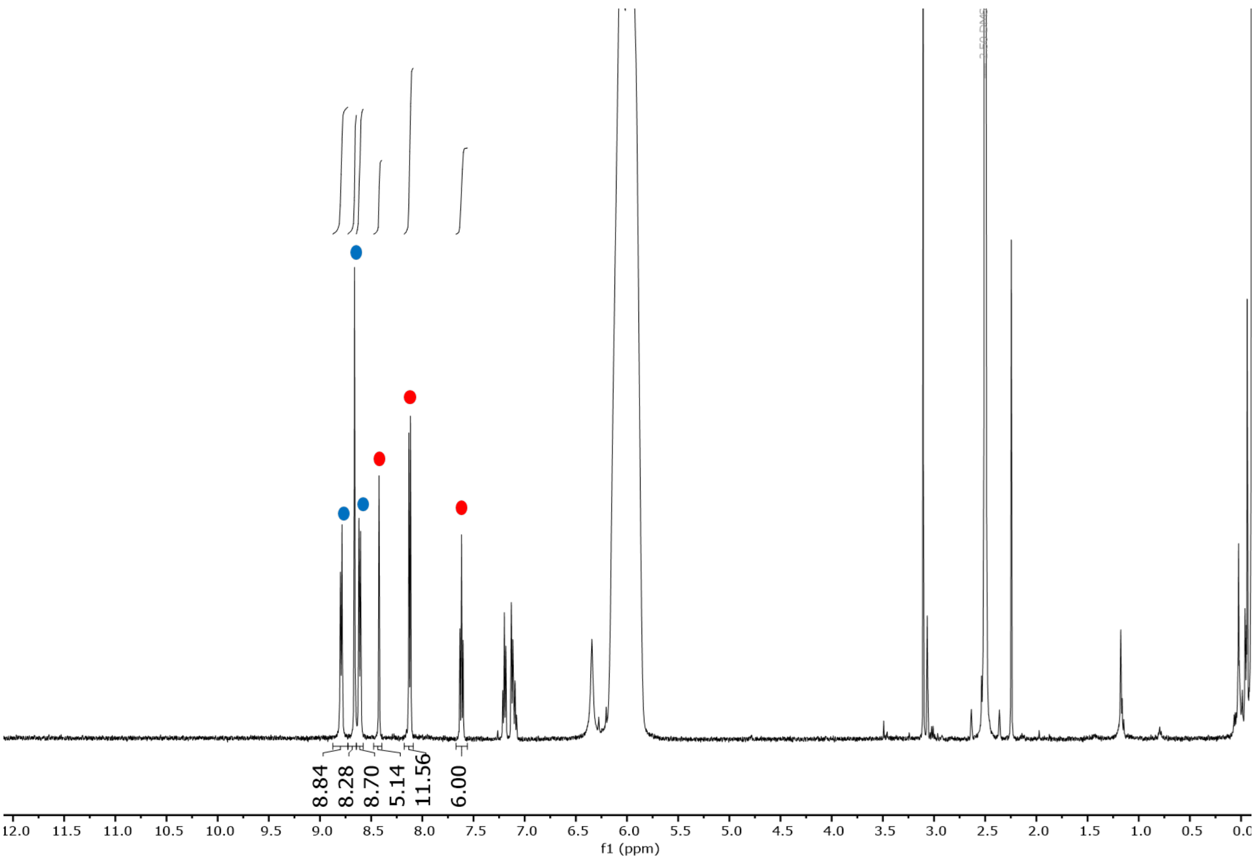

Figure S10: ^{1}HNMR spectra of [Zr*m*BDC][H_2tcpp], digested with 5 drops of DCl in d_6-DMSO. Porphyrin peaks are labeled in blue and cage peaks are labeled in red.

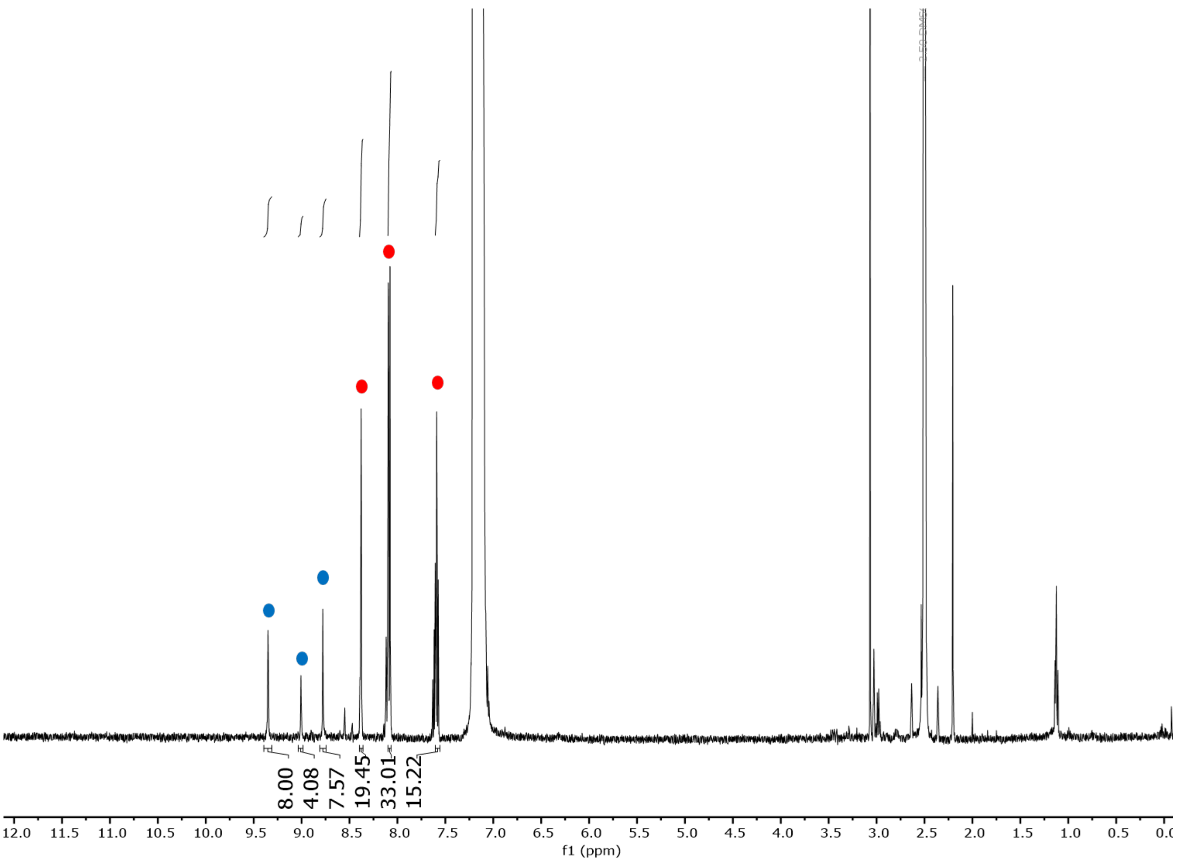

Figure S11: ^{1}HNMR spectra of [Zr*m*BDC][H_2ocpp], digested with 5 drops of DCl in d_6-DMSO. Porphyrin peaks are labeled in blue and cage peaks are labeled in red.

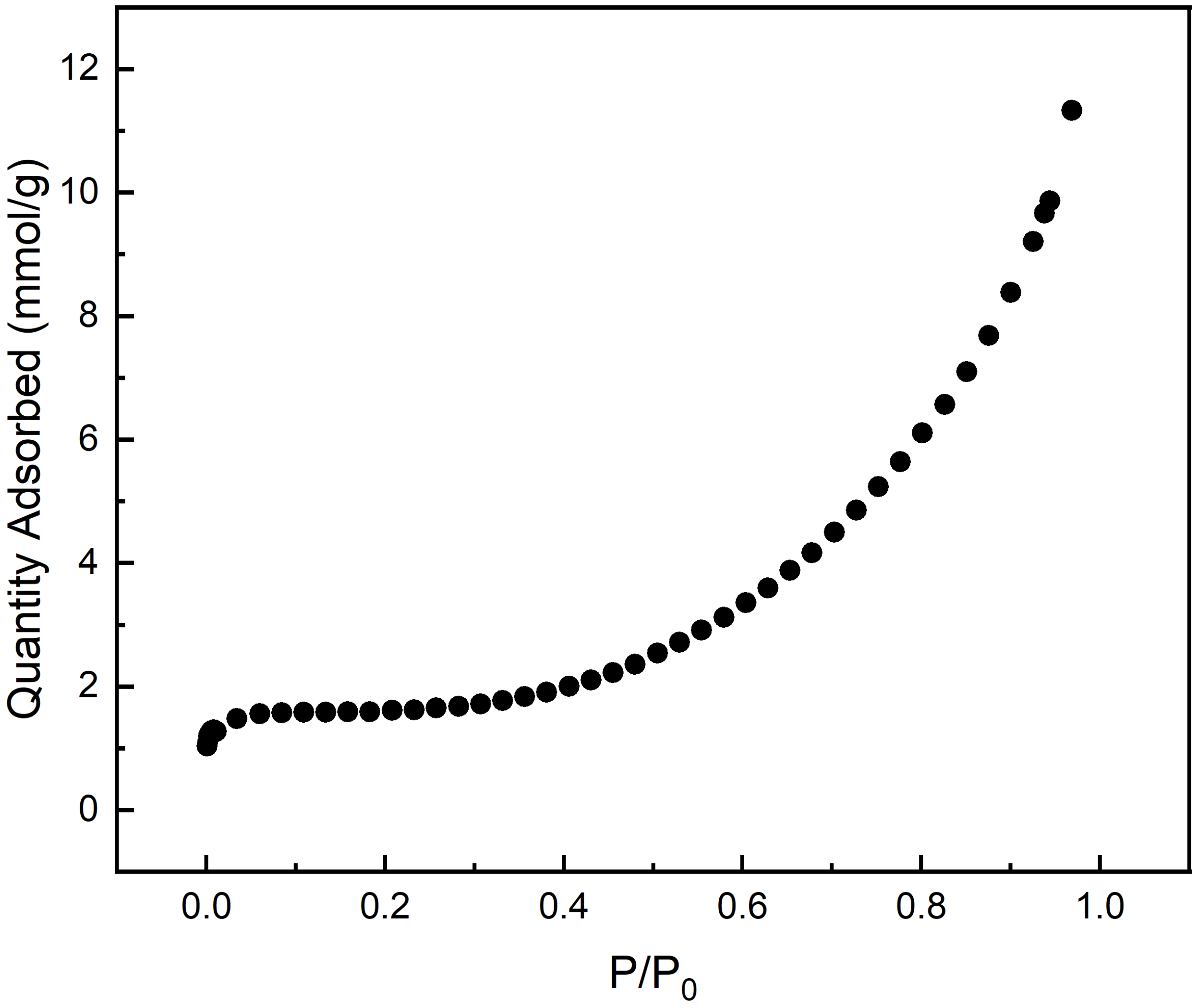

Figure S12: Isothermal gas adsorption measurement for [Zr*m*BDC][H_2tcpp] using N_2 at 77 K. The N_2 accessible surface area (200 m^2/g) was calculated using the Langmuir model.

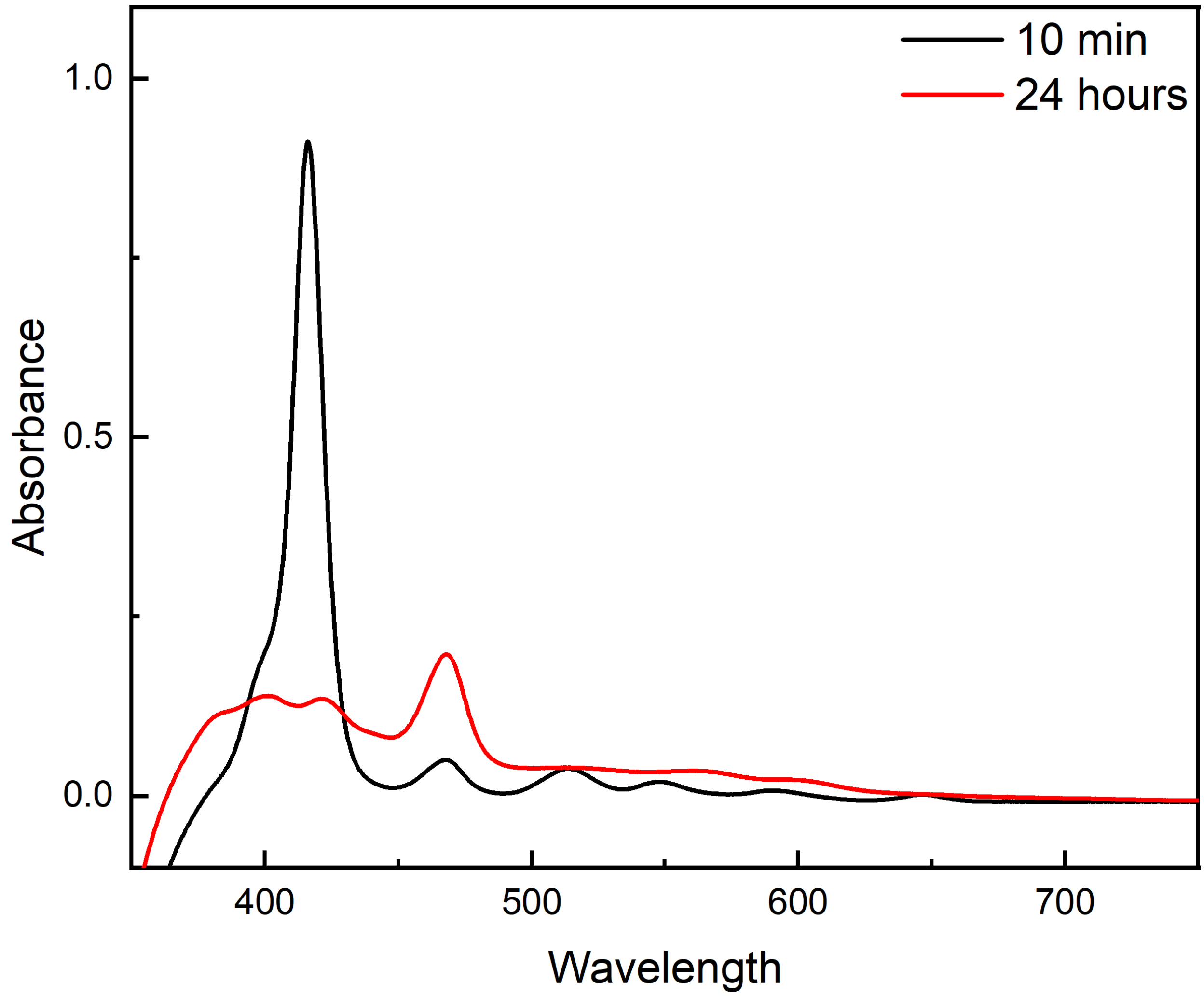

Figure S13: UV-vis plot of the metalation of a [Zr*m*BDC][H_2(tcpp)] film with $MnCl_2$ in MeOH.

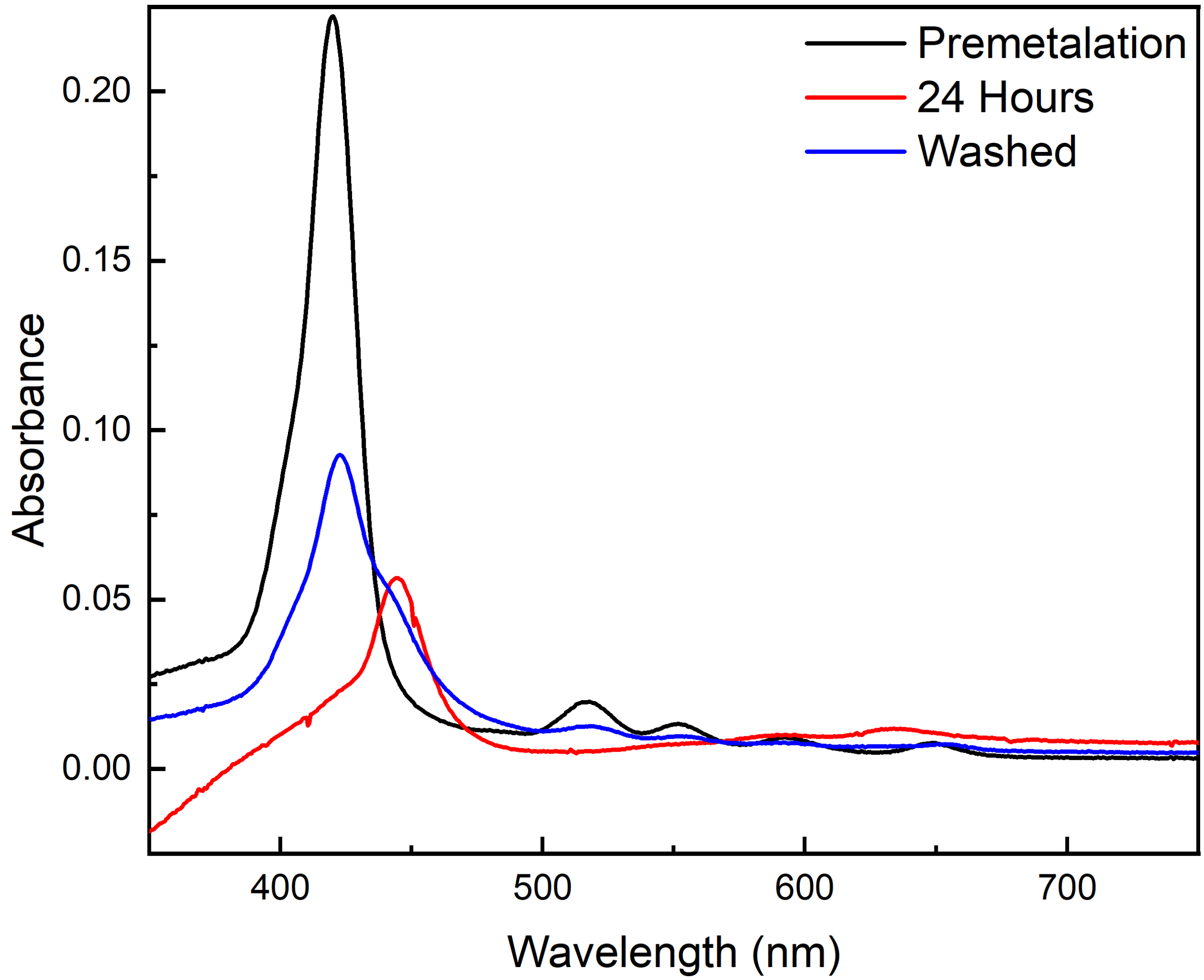

Figure S14: UV-vis plot of the metalation of a [Zr*m*BDC][H_2(tcpp)] film with $MnCl_2$ in THF and subsequent demetallation upon washing with fresh THF.

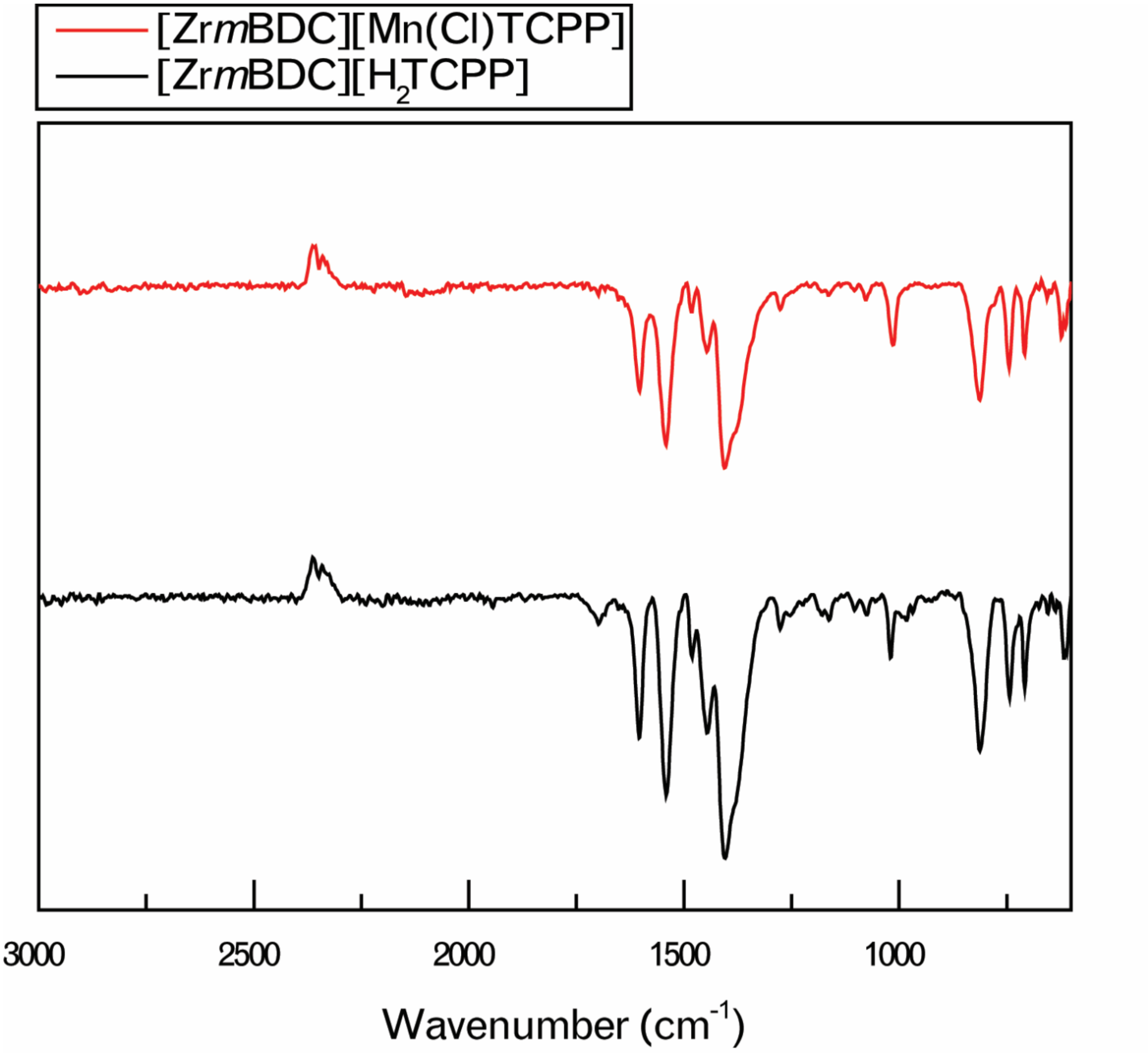

Figure S15: Comparison of the IR spectra of [Zr*m*BDC][H_2TCPP] (bottom) with that of [Zr*m*BDC][Mn(Cl)TCPP] (top).

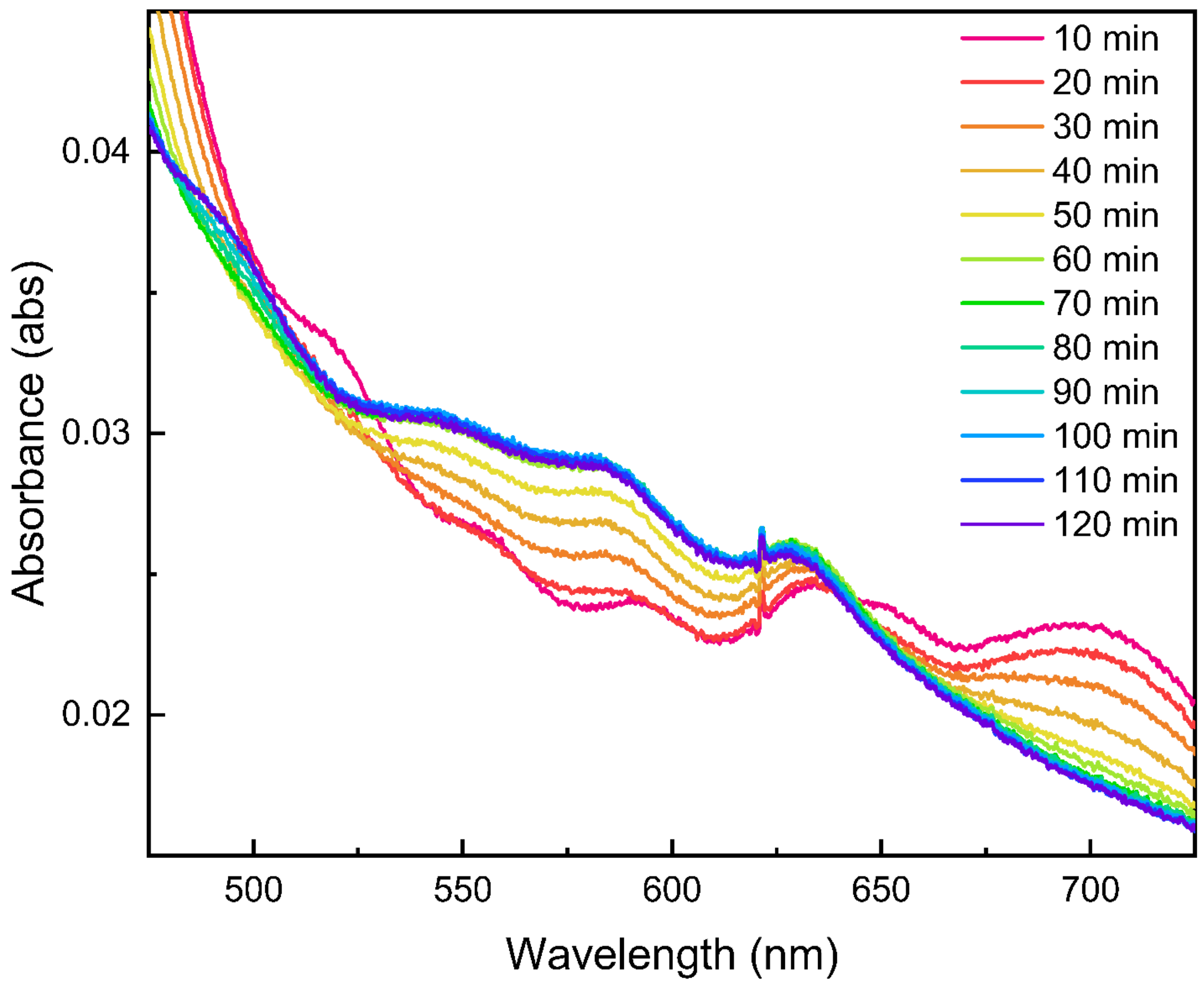

Figure S16: Time-dependent plot of the porphyrin Q-bands during the metalation of a 10 cycle [Zr*m*BDC][H_2(tcpp)] film with $Mn(HMDS)_2$.

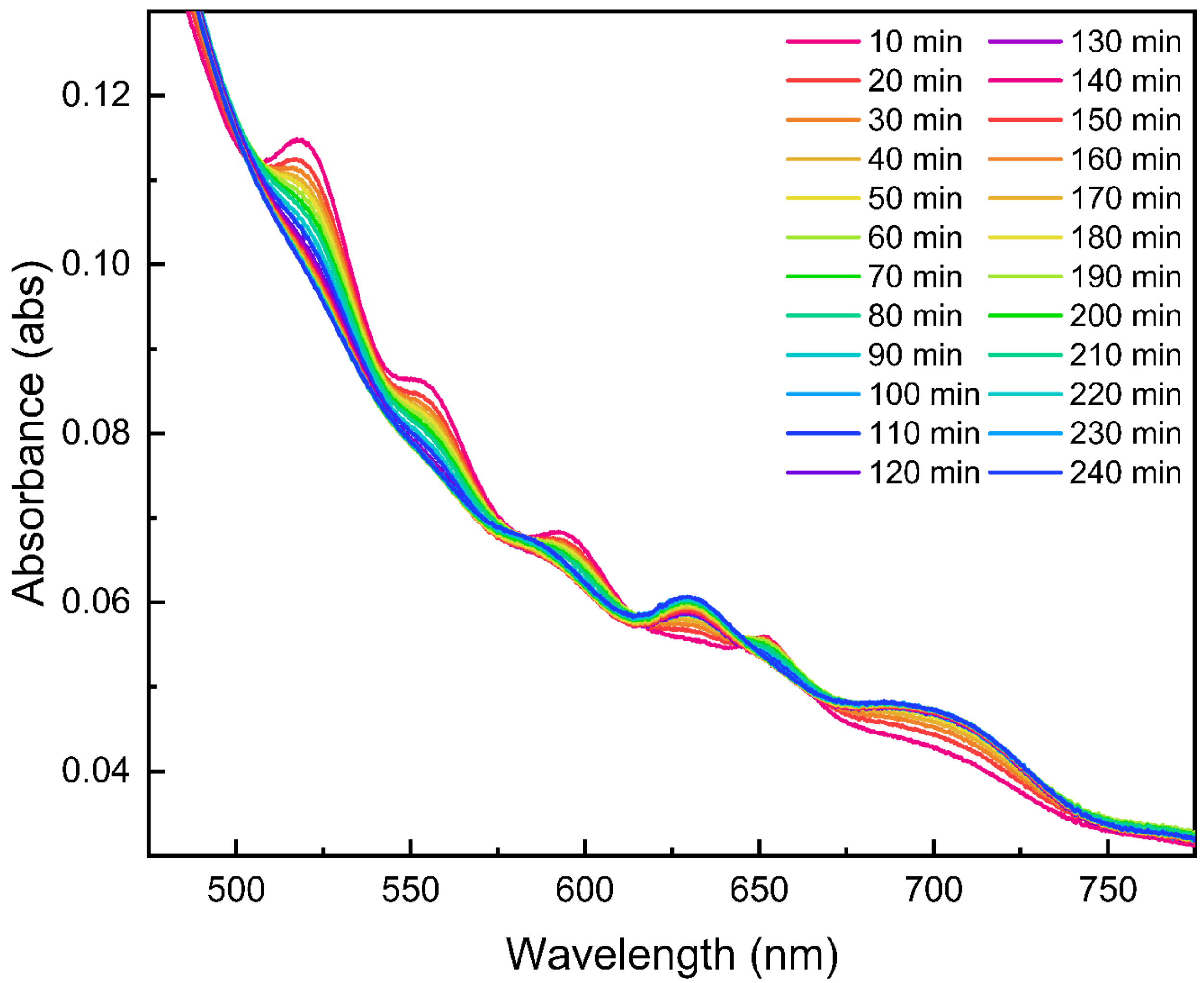

Figure S17: Time-dependent plot of the porphyrin Q-bands during the metalation of a 20 cycle [Zr*m*BDC][H_2(tcpp)] film with $Mn(HMDS)_2$.

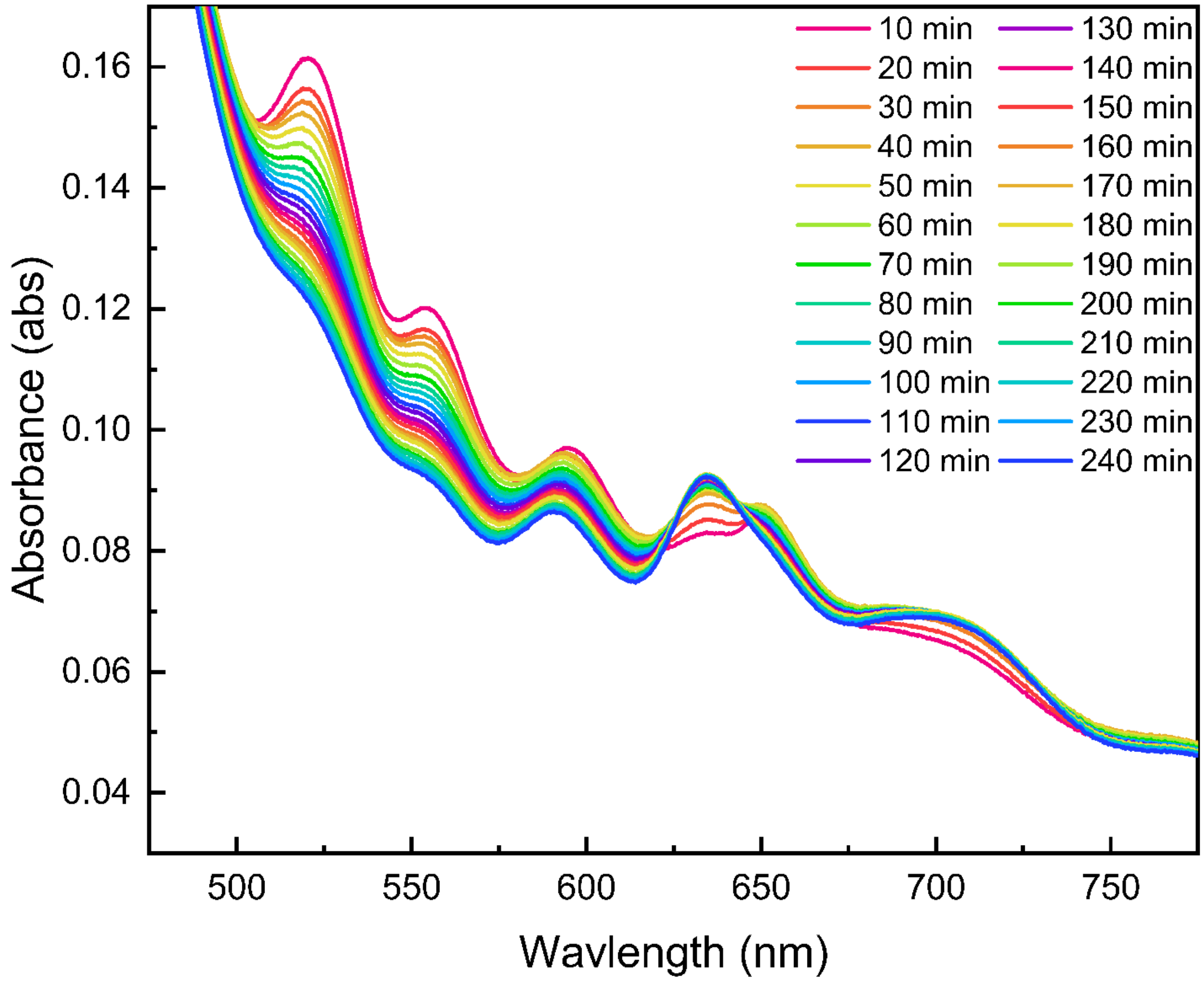

Figure S18: Time-dependent plot of the porphyrin Q-bands during the metalation of a 30 cycle [Zr*m*BDC][H_2(tcpp)] film with Mn(HMDS)$_2$.

References

(1) A. J. Gosselin, A. M. Antonia, K. J. Korman, M. M. Deegan, G. P. A. Yap, E. D. Bloch, *J. Am. Chem. Soc.*, 2021, **143**, 14956–14961.

(2) A. J. Gosselin, G. E. Decker, B. W. McNichols, J. E. Baumann, G. P. A. Yap, A. Sellinger, E. D. Bloch, *Chem. Mater.*, 2020, **32**, 5872–5878.

(3) S. Du, X. Yu, G. Liu, M. Zhou, E.-S. M. El-Sayed, Z. Ju, K. Su, D. Yuan, *Cryst. Growth Des.*, 2021, **21**, 692–697.

(4) Z. Abada, L. Ferrie, B. Akagah, A. T. Lormier, B. Figadere, *Tetrahedron Lett.*, 2011, **52**, 3175–3178.

(5) X. Zhao, L. Yuan, Z.-q. Zhang, Y.-s. Wang, Q. Yu, J. Li, *Inorg. Chem.*, 2016, **55**, 5287–5296.

(6) A. D. Cardenal, H. J. Park, C. J. Chalker, K. G. Ortiz, D. C. Powers, *Chem. Commun.*, 2017, **53**, 7377–7380.

(7) W. Fudickar, J. Zimmermann, L. Ruhlmann, J. Schneider, B. Order, U. Siggel, J.-H. Fuhrhop, *J. Am. Chem. Soc.*, 1999, **121**, 9539–9545.

(8) H. Burger, U. Wannagt, *Monatshefte fur Chemie*, 1964, **95**, 1099-1102.

(9) J. D. Simmons, A. Sur, A. A. Ezazi, K. J. Korman, S. Sarkar, E. T. Iverson, E. D. Bloch, D. C. Powers, *J. Am. Chem. Soc.*, 2023, **145**, 25068–25073.
